# Fishes of the Conambo River Basin, an unexplored area in the Ecuadorian Amazon: first annotated checklist

**DOI:** 10.7717/peerj.21003

**Published:** 2026-03-24

**Authors:** Jonathan Valdiviezo-Rivera, Fernando Sanchez, Daysi Gualavisi-Cajas, Fredy Nugra, Fernando Anaguano-Yancha, Betsy Chango, Jeaneth Mashian, Blanca Ríos-Touma, Gabriela Echevarria

**Affiliations:** 1 Instituto Nacional de Biodiversidad, Quito, Pichincha, Ecuador; 2 Universidad de Las Américas, Facultad de Ciencias Aplicadas, BIOMAS, Campus UDLAPark, Quito, Pichincha, Ecuador; 3 Wildlife Conservation Society, Programa Ecuador, Quito, Pichincha, Ecuador; 4 Confederación de Nacionalidades Indígenas de la Amazonía Ecuatoriana, Puyo, Pastaza, Ecuador; 5 World Wildlife Fund, Quito, Pichincha, Ecuador; 6 Colegio de Ciencias Biológicas y Ambientales (COCIBA), Global Research & Solutions Center, Universidad San Francisco de Quito, Quito, Pichincha, Ecuador

**Keywords:** Freshwater fish species inventory, Indigenous fishing practices, Neotropical fish, Taxonomy

## Abstract

The ichthyofauna of the Ecuadorian portion of the Marañón drainage remains poorly documented, particularly in the tributaries of the Tigre River. Here, we provide the first consolidated dataset of freshwater fish species from the Conambo River, including voucher-based records, locality information, habitat and hydrological context, fisheries practices, and photographic documentation. The dataset comprises 118 species representing seven orders and 31 families, including taxa not previously recorded for Ecuador and specimens that may represent undescribed species. A complementary dataset documents fish species captured by Shiwiar and Sapara fishers, providing additional information on locally important taxa and subsistence practices. Together, these datasets offer a new baseline for taxonomic, biogeographic, and ecological research in one of the least studied regions of the Ecuadorian Amazon and support future efforts to document and understand freshwater biodiversity in the Marañón drainage.

## Introduction

The Neotropical region is home to a spectacular diversity of fish, unsurpassed by any other biogeographic region on the planet (Pelicice et al., 2021). More than 6,000 freshwater species have been formally described and cataloged (Albert, Tagliacollo & Dagosta, 2020). This figure is certainly incomplete, as estimates indicate the existence of between 8,000 and 9,000 species in South America alone (Reis et al., 2016). Indeed, over the last ten years, various efforts have been made to conduct rapid biological inventories, which have enabled the recording of the diversity of flora and fauna in several Amazonian areas.

For the Amazon Drainage, 2,406 species of freshwater fish have been registered (Jézéquel et al., 2020a; Jézéquel et al., 2020b), of which 1,043 are endemic species (Jézéquel et al., 2020a; Jézéquel et al., 2020b). The vast heterogeneity of environmental conditions, including climatic and hydrological gradients, in combination with habitat sizes, has favored this high fish diversity (Oberdorff et al., 2019). Unfortunately, the expansion of anthropogenic activities, such as agriculture, oil exploration, mining, and dam construction, in combination with overfishing to supply commercial and ornamental markets, are threatening freshwater fish faunas through habitat modification, pollution and destruction (Roach et al., 2013; Azevedo-Santos et al., 2016; Anderson et al., 2018; Aguirre et al., 2021).

Nevertheless, for some Amazonian countries, such as Ecuador, the inventory of the ichtyofauna remains incomplete. In this region, more than 700 freshwater fish species have been registered (Aguirre et al., 2021). However, the list of freshwater fish species for Ecuador and the Ecuadorian Amazon dates from 2012 (Barriga, 2012), and thus, it is not up to date. Inventories at the basin level remain scarce and are concentrated in the northern area, along the Napo River (Valdiviezo-Rivera, Carrillo-Moreno & Gea-Izquierdo, 2017; Escobar-Camacho et al., 2025). Furthermore, the ichthyofauna of the Marañón Drainage in Ecuador has been poorly documented, and studies have concentrated on the Pastaza River (Rivadeneira, Anderson & Dávila, 2010). Thus, the threats faced by fish habitats in the Amazon Drainage highlight the urgent need for more detailed inventories of the ichthyofauna.

The Conambo River is one of these less-explored rivers within the Ecuadorian Amazon. To our knowledge, the information about the ichthyofauna and fisheries of this river is nonexistent. The Conambo is located in the lowland tropical rainforest of the Ecuadorian Amazon. It joins the Pindo River at the border with Peru to become the Tigre River, which flows into the Marañón River and ultimately into the Amazon River (Bowser, 2000). This area is located on the border with Peru, which, due to its difficult access and remoteness, presents a poor ichthyological collection (Jézéquel et al., 2020a; Jézéquel et al., 2020b).

Here, we present the first curated dataset of freshwater fish species recorded in the Conambo River Basin, including validated taxonomic information, nomenclatural authorities, specimen records, and detailed metadata for each occurrence. This dataset provides the first openly accessible documentation of the ichthyofauna of the Conambo River and represents a baseline resource for future studies in taxonomy, biodiversity assessment, and conservation planning in the central Ecuadorian Amazon. We also provide a dataset on the fishing practices by the Shiwiar and the Sapara nationalities in the Conambo River. By making these records publicly available, we aim to facilitate further research and comparative analyses across the Marañón drainage and adjacent basins.

## Materials & Methods

### Study area

The Conambo River basin, located in the southern region of the Ecuadorian Amazon, is part of the Tigre River basin. The Conambo River represents an exceptionally well-preserved hydrographic system, characterized by its near-pristine condition, due to its remote location and minimal access. This river originates on the Oriental slopes of the Andes, flowing from east to south, spanning 215 km in length and covering an area of 7,316 km^2^ in Ecuador (CISPDR, 2016). This basin is part of the territories of Achuar, Sapara and Shiwiar nations, who live in small communities with traditional agricultural practices that include small orchards with manioc and plantain crops (López & Sierra, 2011). The land use is mainly of tropical wet forest, with a very small proportion dedicated to agriculture (CISPDR, 2016). The territory of the Shiwiar Nationality is located southeast of Pastaza Province, Pastaza Canton, Corrientes River Parish, in the upper basin of the Corrientes and Tigre Rivers. It borders the Sapara Nationality to the north and west, the Achuar Nationality to the south, and the Republic of Peru to the east. It covers an area of 220,456.9 hectares and retains 98.6% of its original forest, making it one of the last remnants of mature primary forest in the Ecuadorian Amazon.

**Figure 1:**
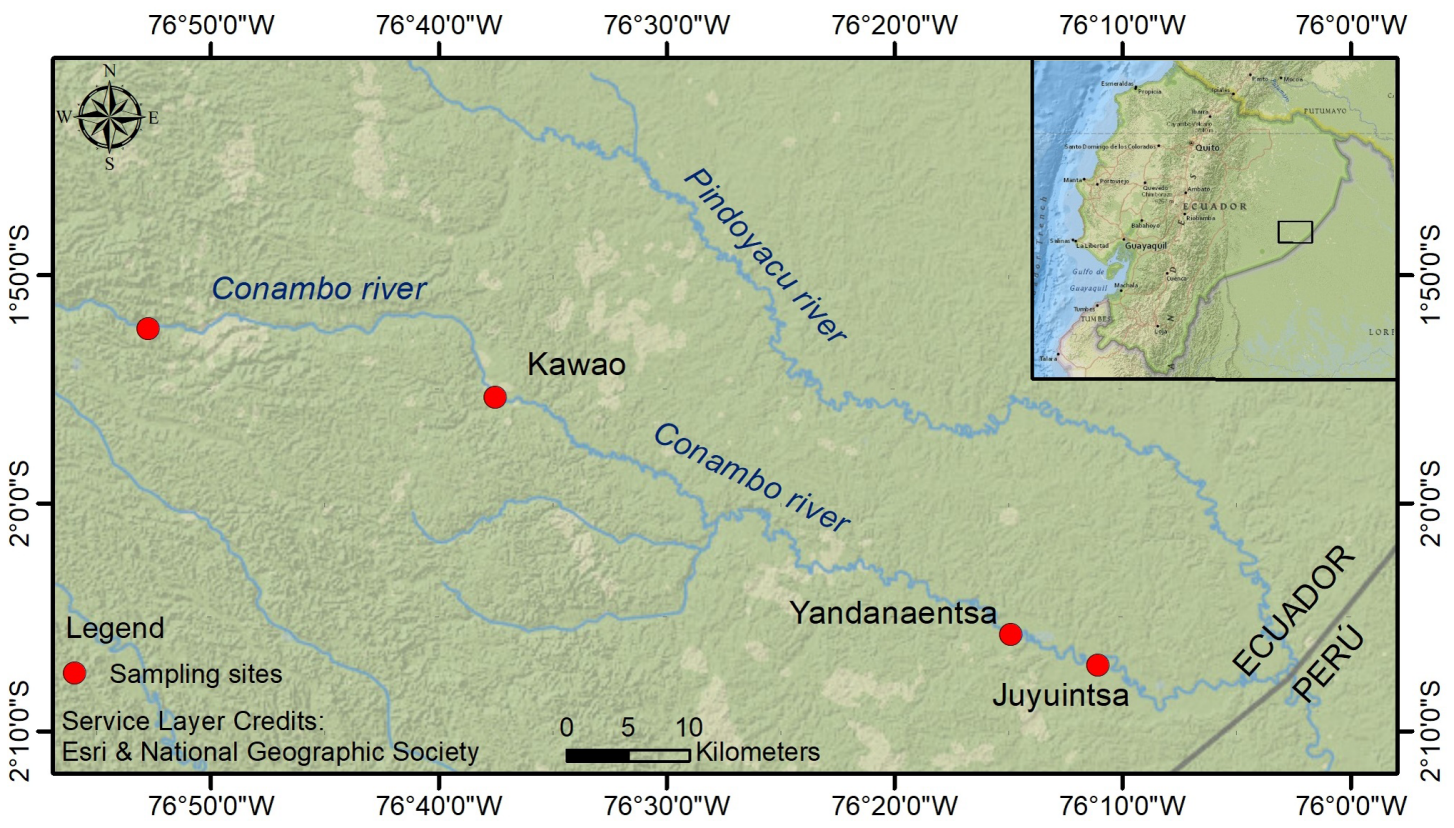
Map of the Conambo River depicting the location of the three longitudinal sites that were sampled in this study. Template: Esri & National Geographic Society. National Geographic World Map basemap. Available at: https://www.arcgis.com, ESRI (2013).

There are marked limnological differences along the Conambo River channel. In the upper reach (Conambo at Kawao community), waters show an average pH of 7.41, specific conductivity of 73.7 µS/cm, and total dissolved solids (TDS) concentration of 45 ppm. A marked shift in water chemistry is observed in the middle reach (Conambo at Yandanaentsa community), where the pH decreases to 6.85, conductivity drops to 39.2 µS/cm, and TDS levels fall to 24.3 ppm. In the lower reach (Conambo at Juyuintsa community), the pH continues to decrease slightly to an average of 6.69, while conductivity increases to 43.4 µS/cm, and TDS to 26.6 ppm. On the contrary, along the channel, dissolved oxygen concentrations remain, ranging from 6.3 to 6.4 mg/L, with saturation levels ranging from 81% to 85%. Likewise, water temperature also shows little longitudinal variation, ranging from 27.4 to 28.3 °C. Samples were carried out in three longitudinal zones of the Conambo River: lower, middle, and low, near the communities Kawao, Yandana Entza, and Juyuintza, respectively (Table 1, Fig. 1). Samples were taken from the Conambo River, its tributaries Nayakim entza, Juyuintza, and other creeks without names.

**Table 1:** Location of the longitudinal zones in the Conambo River where fish samplings were carried out. Geographic coordinates, elevation and Shiwiar communities associated to the three longitudinal zones sampled in this study.

**Number**	**Site codes**	**Longitudinal zone**	**Sites**	**Coordenates utm datum WGS84**		**Elevation** (m)
				**X**	**Y**	
1	Kaw-25	Upper Conambo	Rio Conambo at Kawao	−1.9227342	−76.6253954	227
2	YAN-25	Middle Conambo	Rio Conambo at Yandanaentsa	−2.09616391	−76.2479685	185
3	JUY-25	Lower Conambo	Rio Conambo at Juyuintsa	−2.11852618	−76.18448881	191

### Fish collections

The fishing trips were carried out during two hydrological phases: high water (March) and low water (October), in order to capture seasonal variability in fish occurrence and fishing practices, providing a representative snapshot of community composition and fishery dynamics across the annual flood pulse, despite not covering a full continuous hydrological cycle. The same sampling effort was applied in the three segments of the Conambo River. Fishes were caught using 20 gill nets (1–8 inches), 10 hooks (1/0 to 8/0), four fishing rods, and two hand nets. In each altitudinal zone, gill nets and hooks were left suspended in each water body for 48 h, and they were revised every four hours, while fishing rods and hand nets were employed for four hours in each water body, encompassing one km segments. In small tributaries of the Conambo River, where the use of gill nets was not possible, the roots of the barbasco plant (*Lonchocarpus utilis*) were submerged and shaken as an ancestral fishing technique, in 200 m segments, previously enclosed with gill nets (Fig. S1). In addition, we verbally asked 15 Shiwiar fishers and 48 Sapara fishers about the names of the fish species and about their fishing practices, specifically, the species they caught and the fishing gear they used, the biomass extracted in kilograms per species, the aquatic habitats where the fishery occurred, and the hydrological period. These differences in the number of fishers between nationalities reflect their respective population numbers. The specimens were anesthetized in 10% Lidocaine solution. In the field, fishes were fixed in formaldehyde 10% and subsequently stored in ethanol 75%. We cataloged and deposited of the specimens in the National Institute of Biodiversity INABIO (MECN-DP). All specimens were legally collected under the INABIO’s collection permit MAATE-ARSFC-2024-0411.

### Specimen determinations

Specimens were determined by the coauthors in the field, and the species was corroborated or corrected through a revision with taxonomic keys in the Fish Collection of the National Biodiversity Institute (INABIO) in Quito. The literature review included Géry (1977); Van der Sleen & Lima (2018), Arbour, Salazar & López-Fernández (2014); Lasso et al. (2014), Lasso et al. (2024), Provenzano & Barriga-Salazar (2018), and Galvis et al. (2006). Taxonomic determinations were based on the Eschmeyer’s Catalog of Fishes (Fricke & Eschmeyer, 2025).

### Database and visualization

The dataset compiled for this Data Report consists of a curated and standardized checklist of freshwater fish species recorded in the Conambo River Basin, available as a persistent link to voucher-based records deposited in the SISBio Ecuador platform (https://bndb.sisbioecuador.bio/bndb/). This online resource contains detailed information for each specimen, including:

• INABIO catalogue number.
• Date of fish collection.
• Taxonomic hierarchy (Order, Family, Species).
• Metadata curated by the national biodiversity system, such as the names of the collector and the species determiner.
• The reference coordinates: latitude/longitude (in decimal degrees) of the collection site.
• Elevation above the sea level of the collection site.
• Details on preservation and location of the specimen.
• Record ID.

Thus, all species included in this Data Report have corresponding entries in the SISBio system. We provide an additional CSV file, containing taxonomic information, locality details, and basic metadata for each specimen record, as well as a second CSV file containing information on the fisheries practices of the Shiwiar and Sapara communities of the Conambo River. All species names were validated against the Catalog of Fishes (Fricke, Eschmeyer & Van der Laan, 2019) to ensure consistency and accuracy.

The dataset of the first CSV file with the collection details includes the following fields, besides the location details, date of collection, and taxonomic hierarchy:

• Hydrological season when the fish was collected: high and low waters.
• Zone: longitudinal zone where the specimen was collected: upper, middle, lower.
• Water body: the name of the river or stream where the fish was collected.
• Field ID: code assigned to the fish specimen in the field.
• Shiwiar/Sapara (Kicwha) names: the local names assigned by the communities in their Indigenous language, when available.

The second dataset, presented as an additional CSV file, encompasses fisheries information of the Shiwiar and Sapara nationalities, and includes the following fields:

• Taxonomy: order, family, and species or genus.
• Nationality: practices are described for the Shiwiar and Sapara communities in the Conambo River.
• Fishing gear: equipment or method to catch fish: barbasco, hooks, traps, cast nets, and others.
• Aquatic habitat: refers to where the species is usually caught. Includes the Conambo River, as well as additional creeks, streams, and ponds.
• Season: it refers to the hydrological period when the species is mainly caught: high and low waters.

To provide users with an overview of the dataset, we generated a set of descriptive visualizations that summarize the taxonomic composition and basic structure of the dataset for documentation and reproducibility purposes. A species accumulation curve was generated to assess whether the dataset captures the majority of species collected during the field campaign in the three longitudinal zones that were sampled in the Conambo River, using the R package iNEXT (Hsieh, Ma & Chao, 2016). These visualizations are intended to help readers understand the geographic and taxonomic scope of the dataset and facilitate its reuse. Tables and figures were made using R 4.4.3 (R Core Team, 2025). The annotated checklist, with further details for species identification, is provided in the Results of this manuscript, together with photographic registers of the majority of species included in the datasets.

## Results

**Figure 2:**
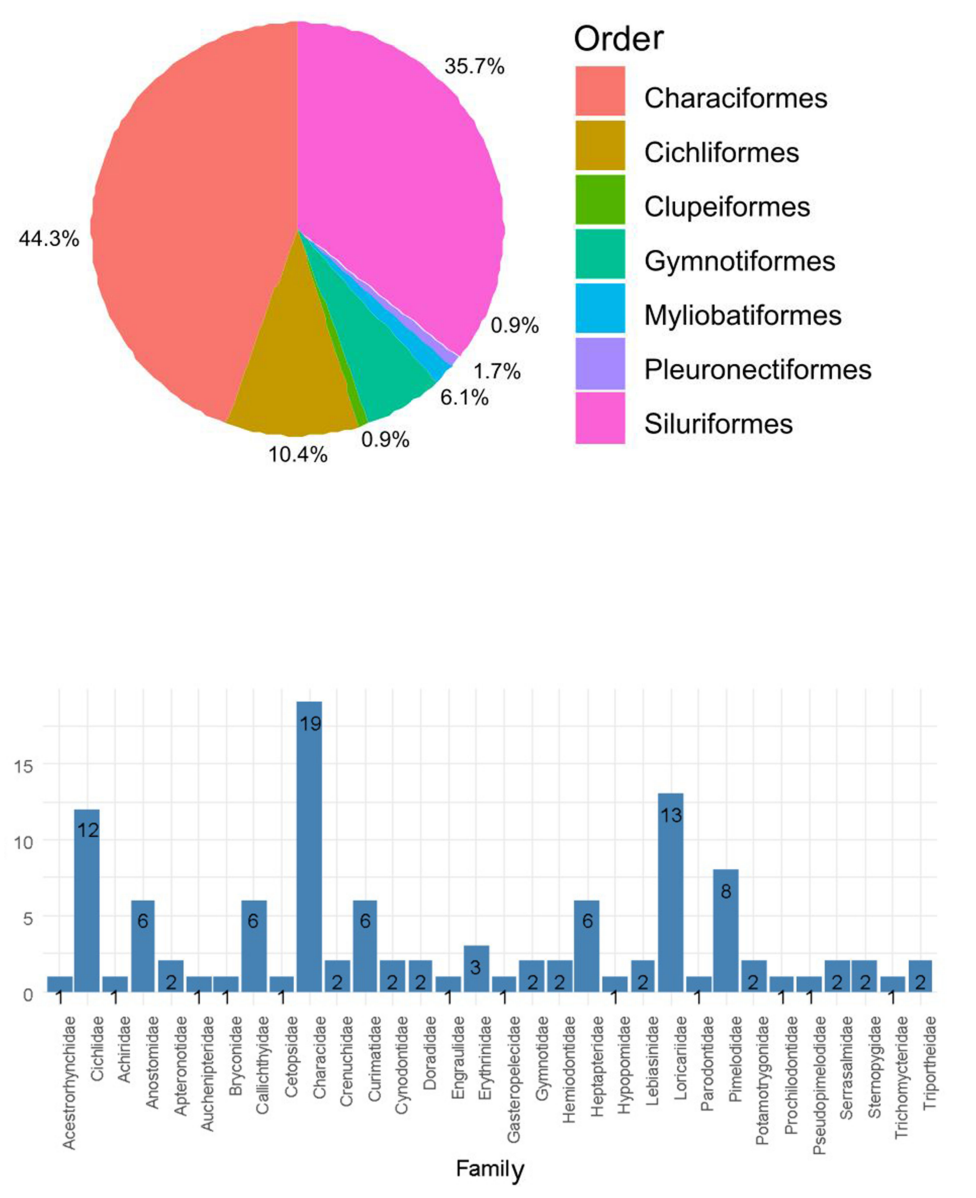
(A) Pie chart showing the proportions of species richness by taxonomic order. (B) Species richness by taxonomic family.

**Figure 3:**
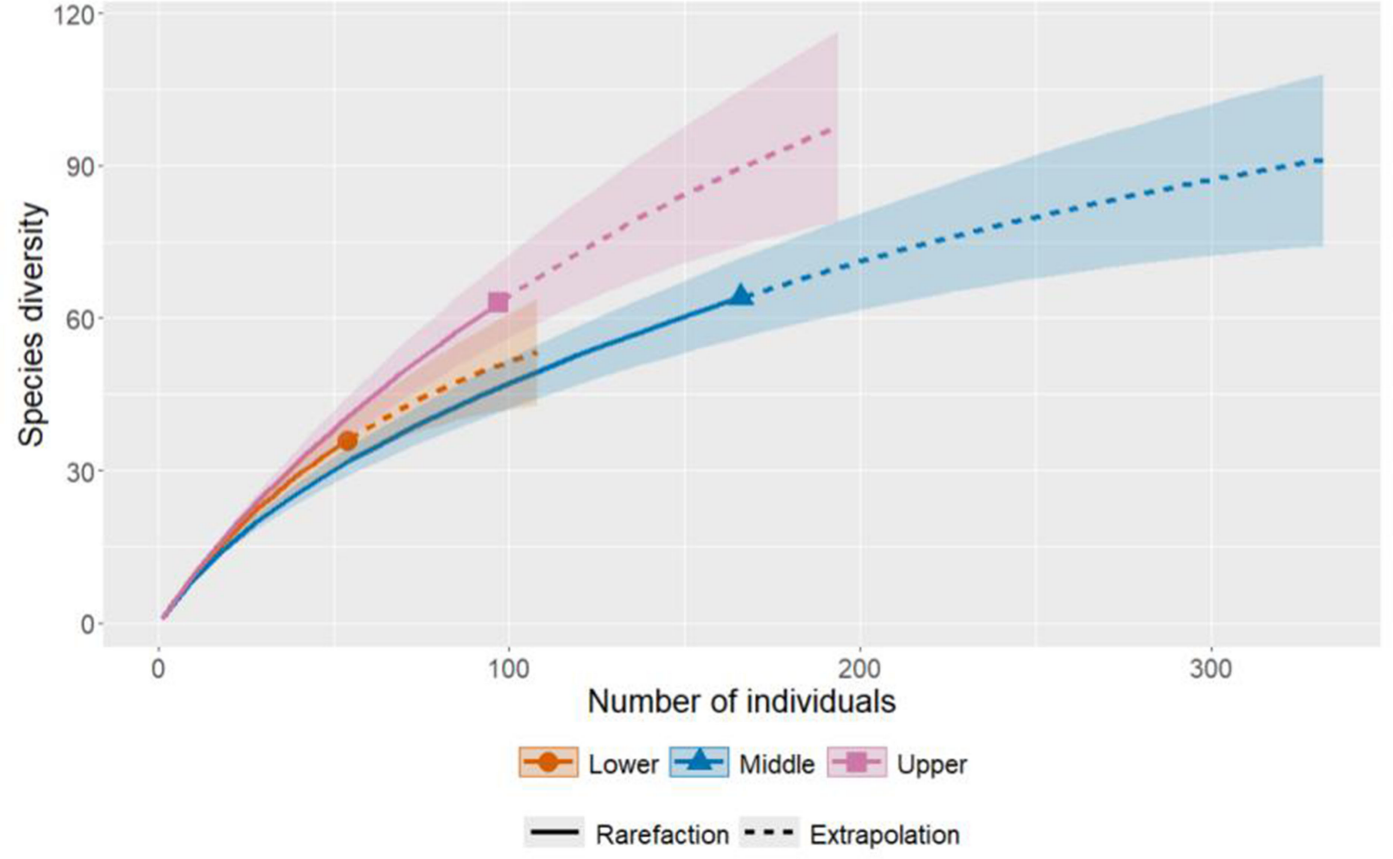
Species accumulation curves by longitudinal zone sampled in the basin of the Conambo River.

A total of 118 freshwater fish species were recorded in the Conambo River Basin, belonging to seven taxonomic orders and 31 families. The ichthyofauna is dominated by Characiformes and Siluriformes (Fig. 2A). The most species-rich families documented in the dataset are Characidae, Loricariidae, and Cichlidae (Fig. 2B). All species occurrences, taxonomic information, localities, and voucher codes are provided in the annotated checklist and in the dataset deposited in SisBio, the website of the National Biodiversity Institute of Ecuador (INABIO) (https://bndb.sisbioecuador.bio/bndb/checklists/checklist.php?clid=15319&pid=0). Species accumulation curves indicated that samples were not complete in any of the three longitudinal zones encompassed in our dataset (Fig. 3).

### Fisheries practices by the Shiwiar and the Sapara

The dataset includes records of fishing practices reported by Shiwiar and Sapara fishers during fieldwork. These records document the fishing gears used, the aquatic environments where they are applied, and the fish species reported for each method. For the Shiwiar, the dataset includes occurrences associated with hook-and-line, cast nets, and the use of *Lonchocarpus utilis* (“barbasco”), the latter mostly applied in small creeks. Species reported by Shiwiar fishers include *Aequidens tetramerus*, *Potamorhina latior*, *Curimata aspera*, *Psectrogaster amazonica*, *Hoplias malabaricus*, *Brycon melanopterus*, *Rhaphiodon vulpinus*, *Serrasalmus gouldingi*, *Prochilodus nigricans*, *Gymnotus carapo*, *Cetopsis coecutiens*, *Oxydoras niger*, *Calophysus macropterus*, *Pimelodus blochii*, *Pseudoplatystoma punctifer*, and *Sorubim elongatus*. Associated fishing locations include the main stem of the Conambo River and adjacent creeks.

For Sapara fishers, the dataset includes records of 33 species across several families, predominantly Pimelodidae and Characidae. According to the dataset, some species (*e.g.*, *Potamotrygon* sp., *Electrophorus multivalvulus*, *Rhaphiodon vulpinus*, *Mylossoma albiscopum*, *Serrasalmus gouldingi*, *Prochilodus nigricans*, *Salminus iquitensis*, *Ancistrus malacops*, and *Panaque* sp.) are associated with records from the main channel of the Conambo River, whereas species such as *Erythrinus erythrinus* and *Creagrutus barrigai* appear in records from smaller creeks. When the dataset includes the season of capture, some species are associated with records during high-water conditions (*e.g.*, *Potamotrygon* sp., *Electrophorus multivalvulus*, *Ancistrus malacops*, *Megalonema platycephalum*), while others appear in records from low-water sampling (*e.g.*, *Erythrinus erythrinus*, *Creagrutus barrigai*, *Charax tectifer*, *Tetragonopterus argenteus*, *Phractocephalus hemioliopterus*). All detailed records, including gear type, locality, and season, are available in the accompanying dataset.

### The annotated fish species check-list of the Conambo River

Here, we present the checklist of the fish species observed in three longitudinal sectors of the Conambo River during October 2024 and March 2025 (Table 2).

**Table 2:** Annotated checklist of fish species in the Conambo River. The taxonomic authority, voucher number, and number of revised specimens are provided for each fish species.

**Species**	**Material examined**
**Order Myliobatiformes**	
**Family Potamotrygonidae**	
*Potamotrygon falkneri Castex & Maciel, 1963*	MECN-DP: 6228 (1 specimen)
*Potamotrygon sp.*	MECN-DP: 6242 (1 specimen)
	MECN-DP: 6256 (1 specimen)
**Order Clupeiformes**	
**Family Engraulidae**	
*Lycengraulis batesii (Günther, 1868)*	MECN-DP: 6331 (1 specimens)
**Order Gymnotiformes**	
**Family Apteronotidae**	
*Apteronotus albifrons* (Linnaeus, 1766)	MECN-DP: 6265 (1 specimens)
*Apteronotus bonapartii* (Castelnau, 1855)	MECN-DP: 6403 (1 specimens)
**Family Sternopygidae**	
*Eigenmannia* sp.	MECN-DP: 6258 (1 specimens)
	MECN-DP: 6410 (1 specimens)
*Sternopygus macrurus* (Bloch & Schneider, 1801)	MECN-DP: 6248 (1 specimens)
	MECN-DP: 6269 (2 specimens)
	MECN-DP: 6283 (1 specimens)
	MECN-DP: 6289 (1 specimens)
**Family Gymnotidae**	
*Electrophorus multivalvulus* Nakashima, 1941	MECN-DP: 6231 (1 specimens)
*Gymnotus carapo* Linnaeus, 1758	MECN-DP: 6249 (2 specimens)
	MECN-DP: 6296 (4 specimens)
	MECN-DP: 6318 (4 specimens)
	MECN-DP: 6319 (3 specimens)
	MECN-DP: 6394 (9 specimens)
**Family Hypopomidae**	
*Brachyhypopomus beebei* (Schultz, 1944)	MECN-DP: 6332 (1 specimens)
**Order Characiformes**	
**Family Crenuchidae**	
*Characidium steindachneri* Cope, 1878	MECN-DP: 6290 (2 specimen)
*Crenuchus spilurus Günther, 1863*	MECN-DP: 6321 (1 specimens)
*Melanocharacidium rex* (Böhlke, 1958)	MECN-DP: 6301 (1 specimen)
**Family Erythrinidae**	
*Erythrinus erythrinus* (Bloch & Schneider, 1801)	MECN-DP: 6237 (2 specimen)
	MECN-DP: 6241 (1 specimen)
	MECN-DP: 6326 (2 specimens)
*Hoplerythrinus unitaeniatus* (Spix & Agassiz, 1829)	MECN-DP: 6252 (1 specimen)
*Hoplias malabaricus* (Bloch, 1794)	MECN-DP: 6239 (2 specimen)
	MECN-DP: 6291 (2 specimen)
	MECN-DP: 6312 (8 specimen)
	MECN-DP: 6316 (2 specimen)
	MECN-DP: 6325 (1 specimen)
	MECN-DP: 6388 (1 specimen)
**Family Parodontidae**	
*Parodon pongoensis (Allen, 1942)*	MECN-DP: 6294 (1 specimen)
**Family Cynodontidae**	
*Cynodon gibbus* (Agassiz, 1829)	MECN-DP: 6353 (2 specimen)
*Rhaphiodon vulpinus* Spix & Agassiz, 1829	MECN-DP: 6377 (1 specimen)
	MECN-DP: 6407 (1 specimen)
**Family Serrasalmidae**	
*Mylossoma albiscopum* (Cope, 1872)	MECN-DP 6411 (1 specimen)
	MECN-DP 6412 (1 specimen)
	MECN-DP 6419 (1 specimen)
*Serrasalmus gouldingi* Fink & Machado-Allison, 1992	MECN-DP 6409 (1 specimen)
**Family Hemiodontidae**	
*Anodus* sp.	MECN-DP 6424 (1 specimen)
*Hemiodus unimaculatus* (Bloch, 1794)	MECN-DP 6235 (1 specimen)
**Family Anostomidae**	
*Abramites hypselonotus* (Günther, 1868)	MECN-DP 6357 (1 specimen)
*Leporinus agassizii* Steindachner, 1876	MECN-DP 6373 (1 specimen)
	MECN-DP 6399 (1 specimen)
	MECN-DP 6400 (1 specimen)
*Leporinus fridereci* (Bloch, 1794)	MECN-DP 6232 (1 specimen)
	MECN-DP 6254 (1 specimen)
	MECN-DP 6363 (1 specimen)
*Leporinus pearsoni* Fowler, 1940	MECN-DP 6358 (1 specimen)
	MECN-DP 6408 (1 specimen)
*Leporinus* sp.	MECN-DP 6421 (1 specimen)
*Schizodon fasciatus* Spix & Agassiz, 1829	MECN-DP 6360 (2 specimen)
	MECN-DP 6361 (1 specimen)
	MECN-DP 6362 (1 specimen)
**Family Curimatidae**	
*Curimata aspera Günther, 1868*	MECN-DP: 6385 (2 specimens)
*Curimatella meyeri* (Steindachner, 1882)	MECN-DP 6379 (1 specimen)
*Potamorhina latior* (Spix & Agassiz, 1829)	MECN-DP 6386 (1 specimen)
	MECN-DP 6387 (1 specimen)
*Psectrogaster amazonica* Eigenmann & Eigenmann, 1889	MECN-DP 6389 (1 specimen)
	MECN-DP 6390 (1 specimen)
*Psectrogaster* sp.	MECN-DP 6422 (1 specimen)
	MECN-DP 6423 (1 specimen)
*Steindachnerina guenteri* Eigenmann & Eigenmann, 1889)	MECN-DP 6259 (3 specimen)
**Family Prochilodontidae**	
*Prochilodus nigricans* Spix & Agassiz, 1829	MECN-DP 6351 (1 specimen)
**Family Lebiasinidae**	
*Pyrrhulina eleanorae* Fowler, 1940	MECN-DP 6345 (2 specimen)
	MECN-DP 6348 (2 specimen)
*Pyrrhulina semifasciata* Steindachner, 1876	MECN-DP 6293 (1 specimen)
	MECN-DP 6327 (5 specimen)
	MECN-DP 6328 (1 specimen)
	MECN-DP 6346 (1 specimen)
**Family Triportheidae**	
*Triportheus angulatus* (Spix & Agassiz, 1829)	MECN-DP 6234 (1 specimen)
	MECN-DP 6378 (1 specimen)
	MECN-DP 6401 (2 specimen)
*Triportheus* sp.	MECN-DP: 6307 (1 specimens)
**Family Gasteropelecidae**	
*Thoracocharax stellatus* (Kner, 1858)	MECN-DP 6253 (2 specimen)
**Family Bryconidae**	
*Brycon melanopterus* (Cope, 1872)	MECN-DP 6233 (1 specimen)
	MECN-DP 6359 (1 specimen)
**Family Characidae**	
*Astyanax bimaculatus* (Linnaeus, 1758*)*	MECN-DP: 6272 (1 specimen)
	MECN-DP: 6299 (1 specimen)
	MECN-DP: 6380 (1 specimen)
*Astyanax* sp.	MECN-DP: 6337 (1 specimen)
*Bario steindachneri* (Eigenmann, 1893)	MECN-DP: 6311 (1 specimen)
*Brachychalcinus nummus* Böhlke, 1958	MECN-DP: 6250 (1 specimens)
*Charax tectifer* (Cope, 1870)	MECN-DP: 6317 (4 specimens)
	MECN-DP: 6329 (1 specimens)
*Chrysobrycon hesperus* (Böhlke, 1958)	MECN-DP: 6315 (1 specimens)
*Creagrutus barrigai* Vari & Harold, 2001	MECN-DP: 6257 (1 specimens)
*Hemigrammus* aff. *ocellifer*	MECN-DP: 6297 (1 specimens)
*Hemigrammus ocellifer* (Steindachner, 1882)	MECN-DP: 6344 (2 specimens)
*Hyphessobrycon agulha* Fowler, 1913	MECN-DP: 6308 (2 specimens)
	MECN-DP: 6338 (6 specimens)
	MECN-DP: 6339 (2 specimens)
*Hyphessobrycon loretoensis* Ladiges, 1938	MECN-DP: 6263 (1 specimens)
*Hyphessobrycon* sp.	MECN-DP: 6304 (3 specimens)
*Galeocharax gulo (Cope, 1870)*	MECN-DP 6282 (1 specimen)
*Knodus gamma* Géry, 1972	MECN-DP: 6287 (1 specimens)
*Knodus megalops* Myers, 1929	MECN-DP: 6274 (2 specimens)
*Moenkhausia comma* Eigenmann, 1908	MECN-DP: 6298 (1 specimens)
*Moenkhausia dichroura* (Kner, 1858)	MECN-DP: 6324 (5 specimen)
*Moenkhausia oligolepis* (Günther, 1864*)*	MECN-DP: 6295 (1 specimen)
	MECN-DP: 6313 (4 specimens)
*Paragoniates alburnus* Steindachner, 1876	MECN-DP: 6243 (1 specimens)
	MECN-DP: 6281 (1 specimens)
*Tetragonopterus argenteus* Cuvier, 1816	MECN-DP: 6354 (1 specimen)
	MECN-DP: 6355 (1 specimen)
**Order Siluriformes**	
**Family Cetopsidae**	
*Cetopsis coecutiens* (Lichtenstein, 1819)	MECN-DP:6273 (1 specimen)
	MECN-DP: 6356 (1 specimen)
	MECN-DP: 6406 (1 specimen)
**Family Trichomycteridae**	
*Vandellia cirrhosa* Valenciennes, 1846	MECN-DP: 6267 (1 specimen)
**Family Callichthyidae**	
*Callichthys callichthys* (Linnaeus, 1758)	MECN-DP: 6381 (2 specimen)
*Corydoras acutus* Cope, 1872	MECN-DP: 6244 (1 specimen)
	MECN-DP: 6292 (2 specimen)
*Corydoras aeneus* (Gill, 1858)	MECN-DP: 6303 (1 specimen)
*Corydoras rabauti*	MECN-DP: 6240 (1 specimen)
*Corydoras zygata* Eigenmann & Allen, 1942	MECN-DP: 6278 (4 specimen)
	MECN-DP: 6320 (2 specimen)
*Megalechis thoracata* (Valenciennes, 1840)	MECN-DP: 6238 (1 specimen)
	MECN-DP: 6285 (1 specimen)
	MECN-DP: 6300 (3 specimen)
	MECN-DP: 6309 (4 specimen)
	MECN-DP: 6310 (1 specimen)
	MECN-DP: 6347 (1 specimen)
**Family Loricariidae**	
*Ancistrus malacops* (Cope, 1872)	MECN-DP: 6286 (2 specimen)
*Ancistrus shuar* Provenzano & Barriga Salazar, 2018	MECN-DP: 6255 (1 specimen)
*Aphanotorulus unicolor* (Steindachner, 1908)	MECN-DP: 6376 (2 specimen)
	MECN-DP: 6382 (1 specimen)
*Farlowella knerii* (Steindachner, 1882)	MECN-DP: 6251 (1 specimen)
*Hypostomus cochliodon*	MECN-DP: 6395 (7 specimen)
	MECN-DP: 6396 (6 specimen)
	MECN-DP: 6413 (1 specimen)
	MECN-DP: 6414 (1 specimen)
*Hypostomus niceforoi* (Fowler, 1943)	MECN-DP: 6405 (1 specimen)
	MECN-DP: 6366 (3 specimen)
*Lasiancistrus heteracanthus* (Günther, 1869)	MECN-DP: 6246 (1 specimen)
*Limatulichthys griseus* (Eigenmann, 1909)	MECN-DP: 6260 (1 specimen)
*Loricaria* cf. *clavipina*	MECN-DP: 6393 (1 specimen)
*Loricaria simillima* Regan, 1904	MECN-DP: 6245 (1 specimen)
	MECN-DP: 6264 (1 specimen)
*Loricariichthys* sp.	MECN-DP: 6279 (1 specimen)
*Panaqolus dentex* (Günther, 1868)	MECN-DP: 6288 (1 specimen)
*Panaqolus nocturnus* (Schaefer & Stewart, 1993)	MECN-DP: 6276 (1 specimen)
	MECN-DP: 6397 (2 specimen)
	MECN-DP: 6398 (4 specimen)
	MECN-DP: 6404 (1 specimen)
	MECN-DP: 6415 (1 specimen)
	MECN-DP: 6416 (1 specimen)
*Panaqolus pantostiktos* Provenzano, Barriga-Salazar & Stewart, 2024	MECN-DP: 6402 (1 specimen)
*Peckoltichthys bachi*	MECN-DP: 6277 (2 specimen)
	MECN-DP: 6375 (1 specimen)
	MECN-DP: 6384 (1 specimen)
*Pseudohemiodon* sp.	MECN-DP: 6392 (1 specimen)
**Family Auchenipteridae**	
*Trachelopterus galeatus* (Linnaeus, 1766)	MECN-DP: 6383 (1 specimen)
**Family Doradidae**	
*Hassar orestis* (Steindachner, 1875)	MECN-DP: 6372 (2 specimen)
*Oxydoras niger* (Valenciennes, 1821)	MECN-DP: 6229 (1 specimen)
**Family Heptapteridae**	
*Pariolius armillatus* Cope, 1872	MECN-DP: 6336 (4 specimen)
*Pimelodella buckleyi* (Boulenger, 1887)	MECN-DP: 6268 (1 specimen)
	MECN-DP: 6341 (1 specimen)
	MECN-DP: 6417 (2 specimen)
	MECN-DP: 6418 (1 specimen)
*Pimelodella gracilis* (Valenciennes, 1835)	MECN-DP: 6284 (1 specimen)
*Pimelodella* sp.	MECN-DP: 6342 (1 specimen)
*Rhamdia quelen* (Quoy & Gaimard, 1824)	MECN-DP: 6349 (2 specimen)
	MECN-DP: 6391 (1 specimen)
**Family Pimelodidae**	
*Calophysus macropterus* (Lichtenstein, 1819)	MECN-DP: 6370 (1 specimen)
*Cheirocerus eques* Eigenmann, 1917	MECN-DP: 6368 (2 specimen)
*Duopalatinus peruanus* Eigenmann & Allen, 1942	MECN-DP: 6371 (1 specimen)
*Hemisorubim platyrhynchus* (Valenciennes, 1840)	MECN-DP: 6364 (1 specimen)
	MECN-DP: 6365 (1 specimen)
*Hypophthalmus oremaculatus* Nani & Fuster, 1947	MECN-DP: 6369 (1 specimen)
*Pimelodus blochii* Valenciennes, 1840	MECN-DP: 6420 (3 specimen)
*Pseudoplatystoma punctifer* (Castelnau, 1855)	MECN-DP: 6230 (1 specimen)
*Sorubim elongatus* Littmann, Burr, Schmidt & Isern, 2001	MECN-DP: 6352 (1 specimen)
**Family Pseudopimelodidae**	
*Microglanis zonatus* Eigenmann & Allen, 1942	MECN-DP: 6275 (2 specimen)
**Order Cichliformes**	
**Family Cichlidae**	
*Aequidens tetramerus* (Heckel, 1840)	MECN-DP: 6306 (1 specimen)
	MECN-DP: 6340 (5 specimen)
	MECN-DP: 6343 (2 specimen)
*Bujurquina* aff. *huallagae*	MECN-DP: 6322 (1 specimen)
	MECN-DP: 6350 (1 specimen)
*Bujurquina mariae* (Eigenmann, 1922)	MECN-DP: 6280 (2 specimen)
*Bujurquina pardus* Arbour, Barriga Salazar & López-Fernández, 2014	MECN-DP: 6305 (1 specimen)
	MECN-DP: 6314 (6 specimen)
*Bujurquina syspilus* (Cope, 1872)	MECN-DP: 6323 (1 specimen)
*Crenicichla lucius* Cope, 1870	MECN-DP: 6333 (1 specimen)
	MECN-DP: 6334 (1 specimen)
	MECN-DP: 6335 (1 specimen)
*Crenicichlla* aff. *sedentaria*	MECN-DP: 6302 (3 specimen)
*Heroina isonycterina* Kullander, 1996	MECN-DP: 6247 (7 specimen)
*Heros efasciatus* Heckel, 1840	MECN-DP: 6266 (1 specimen)
*Laetacara flavilabris (Cope, 1870)*	MECN-DP: 6261 (2 specimen)
	MECN-DP: 6262 (3 specimen)
	MECN-DP: 6330 (1 specimen)
*Satanoperca jurupari* (Heckel, 1840)	MECN-DP: 6367 (1 specimen)
*Saxatilia proteus* Cope, 1872	MECN-DP: 6270 (1 specimen)
	MECN-DP: 6271 (1 specimen)
**Order Pleuronectiformes**	
**Family Achiridae**	
*Hypoclinemus mentalis* (Günther, 1862)	MECN-DP: 6236 (1 specimen)
	MECN-DP: 6374 (1 specimen)

### Material examined

*Potamotrygon falkneri:* Ecuador: MECN-DP 6228, 1, 500 mm SL.

Fig. 4A, (Table 2)

**Figure 4:**
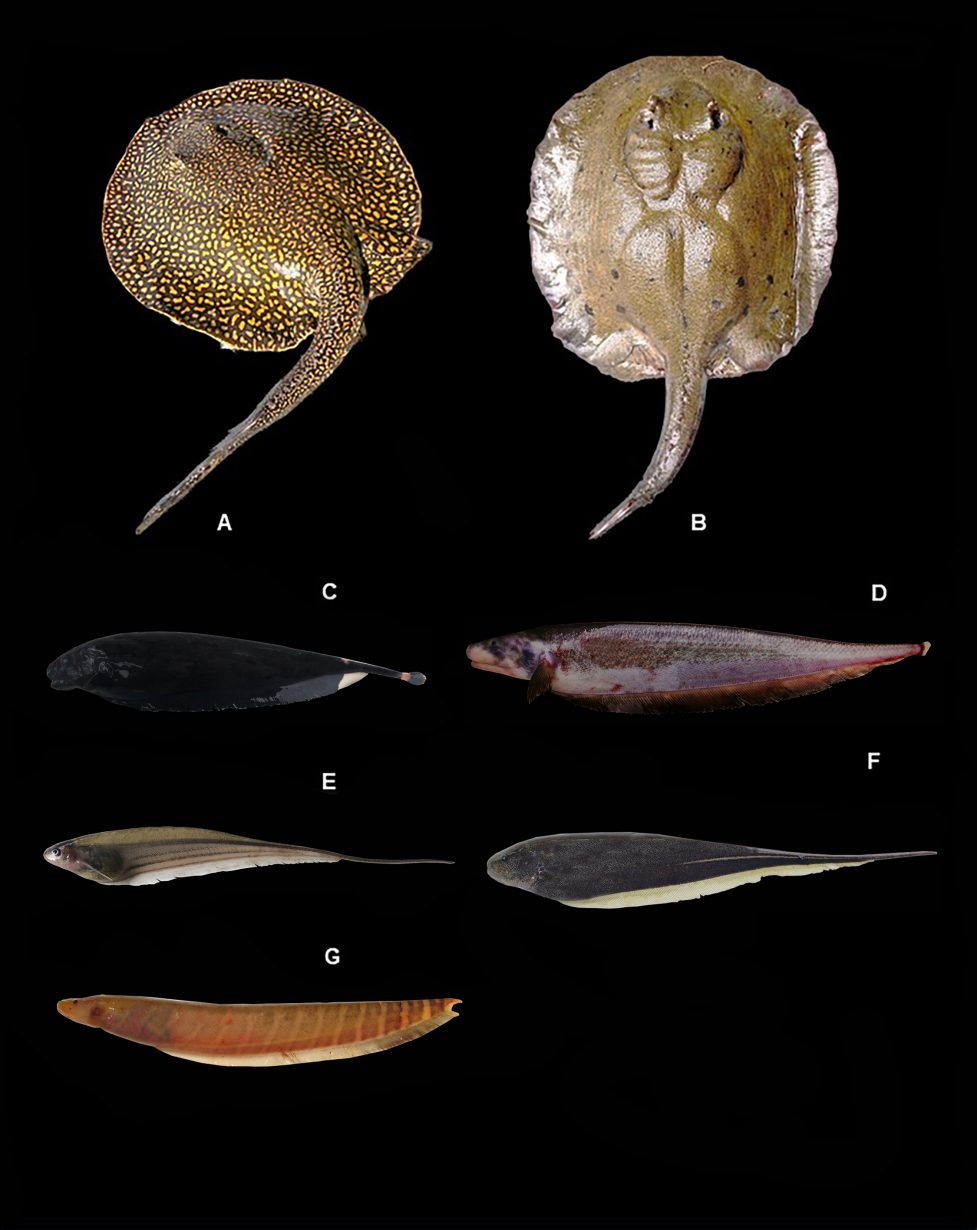
Representative fishes of the Conambo River. Shiwiar and Kichwa names available are included as depicted in the dataset. Shi: name in Shiwiar, Ki: name in Kichwa. (A) *Potamotrygon falkneri*. (B) *Potamotrygon* sp. (Shi: Pushu kashap, Ki: Raya). (C) *Apteronotus albifrons*. (D) *Apteronotus bonapartii* (Shi: Unchiminiar Muuta). (E) *Eigenmannia* sp. (Shi: Churuind wancha). (F) *Sternopygus macrurus* (Shi: Churuindwancha). (G) *Gymnotus carapo* (Shi: Tsunguipit, Ki: Turuyayu).

**Identification.** A cartilaginous fish with a compressed, disc-shaped body. The mouth is ventrally positioned, and the teeth lack prominent cusps. The dorsal surface of the disc is dark brown, with a pattern of light or orange spots in a circular, rosette-shaped, vermicular pattern. The pelvic fins have the same pattern as the disc. The belly is generally pale in color with mottled black spots.

*Potamotrygon* sp.: Ecuador: MECN-DP 6242, 1, 340 mm SL; MECN-DP 6256, 1, 507 mm SL.

Fig. 4B, (Table 2)

**Identification.** A cartilaginous fish with a compressed, disc-shaped body. The mouth is ventrally positioned, and the teeth lack prominent cusps. The dorsal surface of the disc is dark brown, with a pattern of mottled black spots. The pelvic fins have the same pattern as the disc. The belly is generally pale in color with mottled black spots.

*Lycengraulis batesii:* Ecuador: MECN-DP 6331, 1, 70 mm SL.

(Table 2)

**Identification**. A medium-sized fish with a compressed body. The snout is elongated and projects over the lower jaw. The teeth are well-developed, canine-like. The scales are silvery. The body is yellowish with dark bands running longitudinally from the operculum to the caudal peduncle. The caudal fin lobes are slightly yellowish.

*Apteronotus albifrons:* **Ecuador:** MECN-DP 6265, 1, 190 mm SL.

Fig. 4C, (Table 2)

**Identification**. Medium-sized fish with a compressed body. The head has a short, rounded snout and small eyes. The body is deep black with a yellow band on the dorsal side of the head and three yellow bands on the posterior side of the body. The anal fin is very long.

*Apteronotus bonapartii:* **Ecuador:** MECN-DP 6403, 1, 310 mm SL.

Fig. 4D, (Table 2)

**Identification**. A medium-sized fish with a compressed body. The head and snout are noticeably large. The body is dark brown or uniformly black. The anal and pectoral fins are completely black.

*Eigenmannia* sp. **Ecuador:** MECN-DP 6258, 1, 130 mm SL; MECN-DP 6410, 1, 159 mm SL.

Fig. 4E, (Table 2)

**Identification**. Elongated and compressed body. The eye size is small in relation to the head length. The skin is almost transparent and yellow in color. It has dark longitudinal lines on the lateral region of the body: one above the lateral line, another at the base of the anal fin, and one between the two. The anal fin is hyaline.

*Sternopygus macrurus:* **Ecuador**: MECN-DP 6248, 1, 160 mm SL, MECN-DP 6269, 2, 75–109 mm SL, MECN-DP 6283, 1, 50.4 mm SL, MECN-DP 6289, 1, 615 mm SL.

Fig. 4F, (Table 2)

**Identification**. Conical snout. Dark brown body with a white longitudinal stripe along the posterior base of the anal fin. Anal fin hyaline.

*Electrophorus multivalvulus*: **Ecuador:** MECN-DP 6231, 1, 740 mm SL.

(Table 2)

**Identification**. Eel-like body, with a very long anal fin that reaches the end of the body, a broad, depressed head, and small, skin-covered eyes. Conical teeth. A dark brown body with mottled white spots.

*Gymnotus carapo:* **Ecuador:** MECN-DP 6249, 2, 320–340 mm SL; MECN-DP 6296, 4, 437–102.7 mm SL. MECN-DP 6318, 4, 127–225 mm SL; MECN-DP 6319, 3, 145–920 mm SL; MECN-DP 6394, 9, 50–185 mm SL.

Fig. 4G, (Table 2)

**Identification**. The body is elongated, compressed, and the head is flattened, with a prominent lower jaw. The eyes are small and covered with skin. The body is brown in color. It has oblique double bands of dark and light brown along the sides of the body that extend to the base of the anal fin.

*Brachyhypopomus beebei:* **Ecuador:** MECN-DP 6332, 1, 107 mm SL.

(Table 2)

**Identification**. The body is elongated and compressed, with a straight dorsal profile in the anterior half. The mouth is located at an inferior position. The color pattern is yellowish, with small black spots and lines arranged above the lateral line. The tail fins are absent, and the pectoral fins have a rounded posterior margin.

*Characidium steindachneri:* **Ecuador:** MECN-DP 6290, 2, 47–49 mm SL.

Fig. 5B, (Table 2)

**Figure 5:**
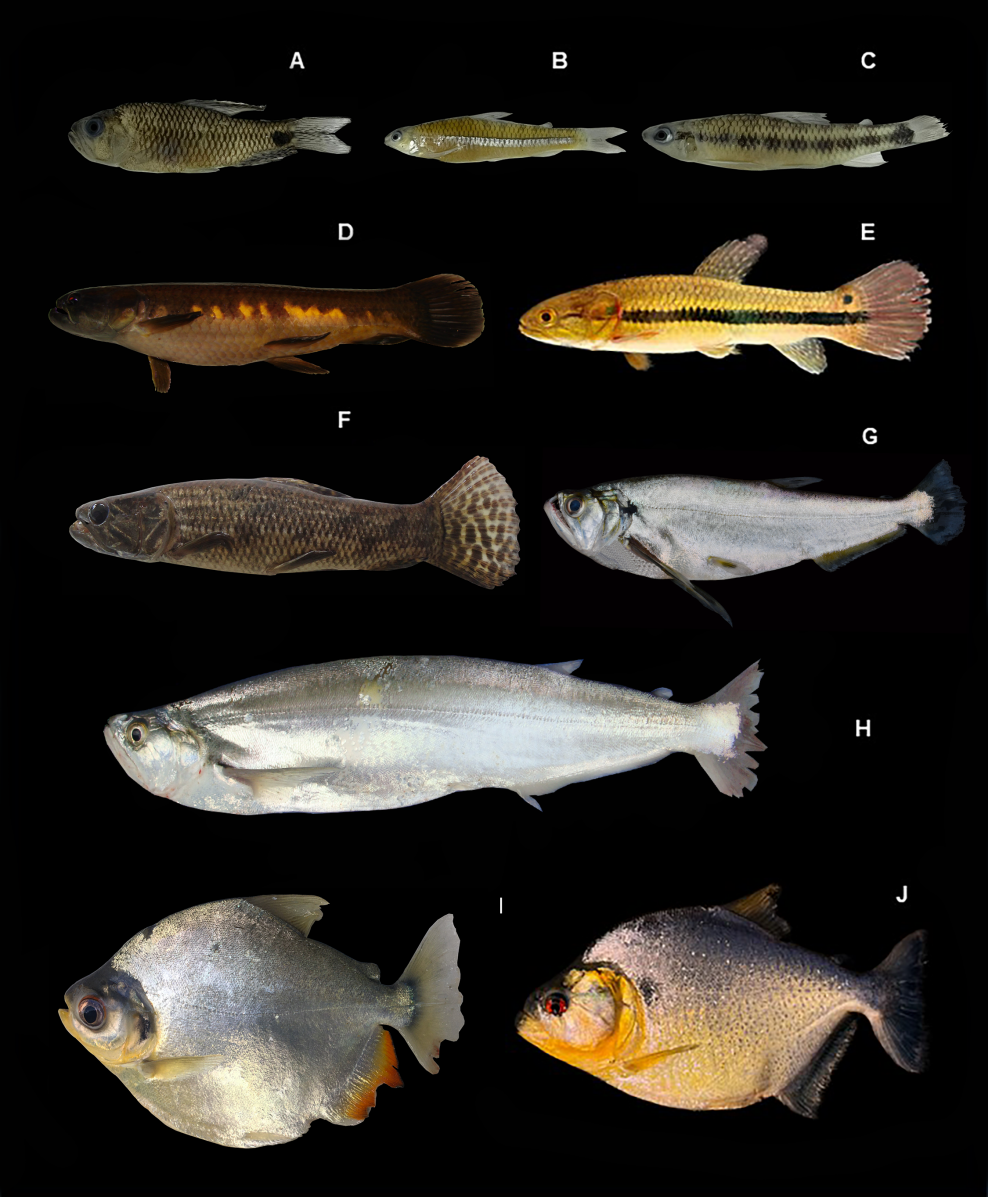
Representative Characiformes of the Conambo River. Shiwiar and Kichwa names available are included as depicted in the dataset. Shi: name in Shiwiar, Ki: name in Kichwa. (A) *Crenuchus spilurus* (Shi: Pakuy Wapuk). (B) *Characidium steindachneri* (Shi: Kusum). (C) *Melanocharacidium rex* (Shi: Kusum). (D) *Erythrinus erythrinus* (Ki: Yayu-Wanchu, Ki: Tariri). (E) *Hoplerythrinus unitaeniatus* (Shi: Kashupiar). (F) *Hoplias malabaricus* (Sh: Kasur, Ki:Pashin). (G) *Cynodon gibbus* (Shi: Shitiu chambirum). (H) *Rhaphiodon vulpinus* (Sh:Manjajchir, Ki: Chambirima). (I) *Mylossoma albiscopum* (Sh: Kapawi, Ki: Capahuari). (J) *Serrasalmus gouldingi* (Ki: Yahuar paña).

**Identification**. Elongated, cylindrical body. Compressed caudal peduncle. Greenish-yellow body. It has a black band extending from the snout to the base of the caudal fin. The dorsal portion of the body has numerous diffuse vertical bands that do not extend beyond the lateral line. Hyaline fins.

*Crenuchus spilurus:* **Ecuador:** MECN-DP 6321, 1, 40 mm SL.

Fig. 5A, (Table 2)

**Identification.** Small fish. Uniformly grayish in color, with an oval black spot located at the base of the caudal peduncle. Lateral line incomplete. Fins reddish. In males, the upper dorsal and anal fins are more developed.

*Melanocharacidium rex:* **Ecuador:** MECN-DP 6301, 1, 38.5 mm SL.

Fig. 5C, (Table 2)

**Identification**. Relatively larger than its conspecifics. Body is elongated and uniform. It has vaguely defined vertical bands. A thin longitudinal band runs from the snout to the caudal peduncle. A rounded dark spot at the base of the caudal fin. The fins are hyaline.

*Erythrinus erythrinus:* **Ecuador:** MECN-DP 6237, 2, 93.9–105.5 mm SL; MECN-DP 6241, 1, 100.3 mm SL; MECN-DP 6326, 2, three mm SL.

Fig. 5D, (Table 2)

**Identification**. Elongated, cylindrical body. The caudal fin is rounded; it lacks an adipose fin. The body coloration is light brown; it has dark bands running from the eye to the posterior edge of the operculum; the dorsal surface of the head has small dark spots.

*Hoplerythrinus unitaeniatus:* **Ecuador:** MECN-DP 6252, 1, 111.5 mm SL.

Fig. 5E, (Table 2)

**Identification**. Cylindrical body. The caudal and anal fins are rounded. The body is brown dorsally and pale yellow on the lower lateral half, with a whitish belly. A dark brown longitudinal band can be distinguished along the midline of the body, running from behind the eye to the base of the caudal median rays. The pectoral and pelvic fins are generally red, while the dorsal, caudal, and anal fins are greenish-brown.

*Hoplias malabaricus:* **Ecuador:** MECN-DP 6239, 2, 551–767 mm SL; MECN-DP 6291, 2, 60–670 mm SL; MECN-DP 6312, 8, 30–80 mm SL; MECN-DP 6316, 2, 420–580 mm SL; MECN-DP 6325, 1, 45 mm SL; MECN-DP 6388, 1, 121 mm SL.

Fig. 5F, (Table 2)

**Identification**. Cylindrical in shape. Brown body with darker diagonal bands. Fins with brown spots forming sinuous lines. Mouth with canine-like teeth.

*Cynodon gibbus:* **Ecuador:** MECN-DP 6353, 2, 190–220 mm SL.

Fig. 5G, (Table 2)

**Identification**. The body is elongated and compressed, with a pronounced ventral keel. It is silvery in color, with a humeral spot and another at the base of the caudal fin. The pectoral fins are highly developed and yellowish-gray with a black spot at their base. The anal fin is very long and hyaline in color. The scales are rough to the touch. The jaw is long with highly developed canine-like teeth that pierce the palate.

*Rhaphiodon vulpinus*: **Ecuador:** MECN-DP 6377,1, 605; MECN-DP 6407, 1, 300 mm SL.

Fig. 5H, (Table 2)

**Identification**. The body is elongated and compressed, darker silvery on the back. It has a jaw with highly developed canine-like teeth. It has tiny scales. The dorsal fin is located in the posterior third of the body.

*Mylossoma albiscopum:* **Ecuador:** MECN-DP 6411, 1, 180 mm SL; MECN-DP 6412,1, 235 mm SL; MECN-DP 6419, 1, 165 mm SL.

Fig. 5I, (Table 2)

**Identification**. Compressed, disc-shaped body. The dorsal shape is concave behind the head and convex between the head and dorsal fin; the premaxilla projects forward. It is uniformly silvery in color. It has a highly developed serrated keel that extends to the anal opening. The anal fin is elongated.

*Serrasalmus gouldingi*: **Ecuador:** MECN-DP 6409, 1, 248 mm SL.

Fig. 5J, (Table 2)

**Identification**. Compressed, disc-shaped body. The dorsal shape is concave behind the head and convex between the head and dorsal fin; the premaxilla projects forward. It is uniformly silvery in color. It has a highly developed serrated keel that extends to the anal opening. The anal fin is elongated.

*Anodus* sp.: **Ecuador:** MECN-DP 6424, 1, 242 mm SL.

Fig. 6A, (Table 2)

**Figure 6:**
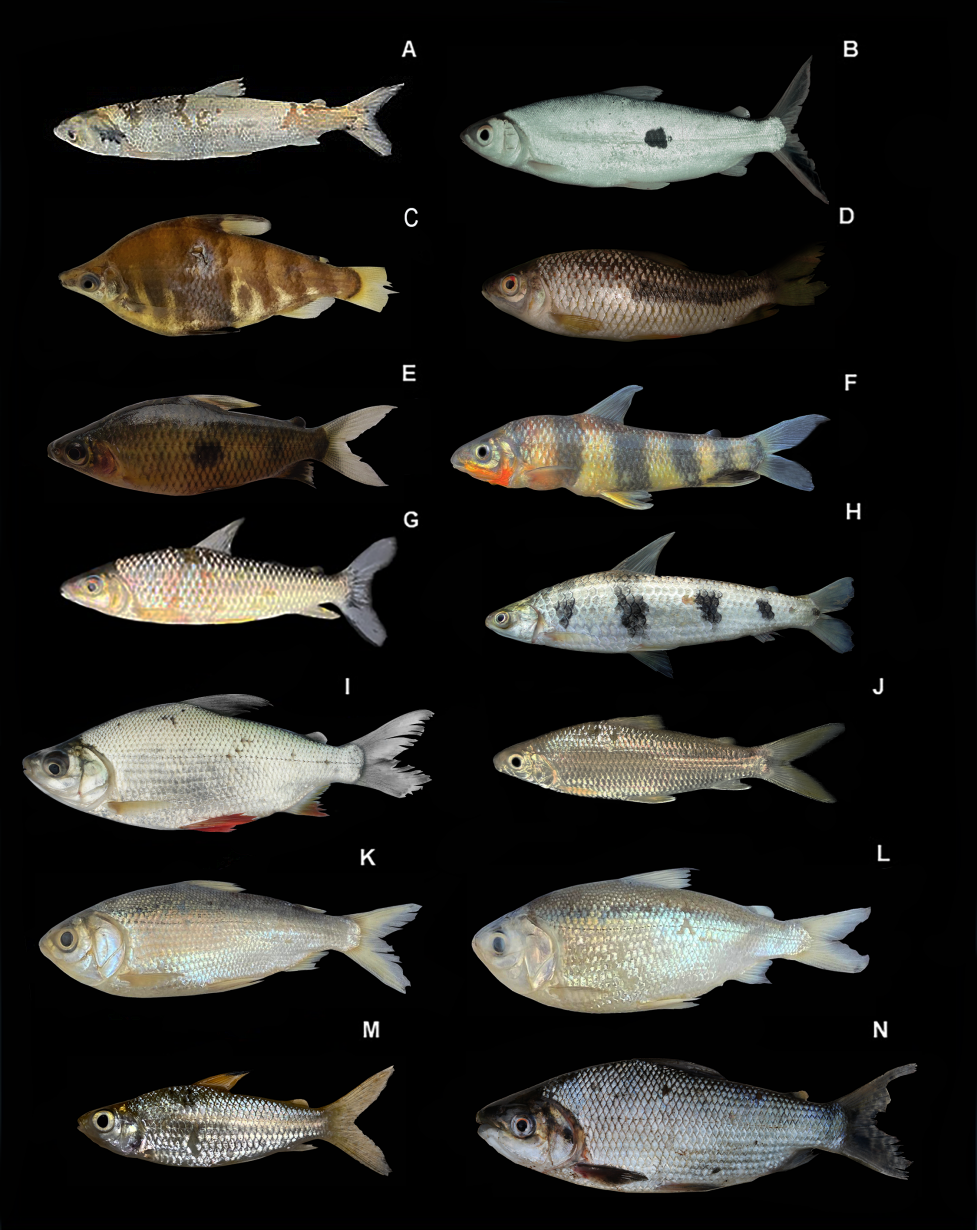
Representative Characiformes of the Conambo River. Shiwiar and Kichwa names available are included as depicted in the dataset. Shi: name in Shiwiar, Ki: name in Kichwa. (A) *Anodus* sp. (B) *Hemiodus unimaculatus* (Shi: Tserengash). (C) *Abramites hypselonotus* (Shi: Nayakim Puutu). (D) *Leporinus agassizii*. (E) *Leporinus friderici* (Shi: Pirum, Ki: Tanla). (F) *Leporinus pearsoni* (Shi: Najarip). (G) *Leporinus* sp. (Shi: Najarip). (H) *Schizodon fasciatus* (Shi: Pirum, Ki: Tanla). (I) *Curimata aspera* (Shi: Apukum, Ki: Kucha challwa). (J) *Curimatella meyeri* (Shi: Kuum, Ki: Sara challwa). (K) *Potamorhina latior* (Ki: Yahuarachi). (L) *Psectrogaster amazónica* (Shi: Apukum, Ki: Kara sapa). (M) *Steindachnerina guentheri* (Shi: Apukum, Ki: Sara challwa). (N) *Prochilodus nigricans* (Shi: Kuum, Ki: Challhwa).

**Identification**. Fusiform body. Silvery in color, slightly darker on the back. Lower terminal mouth more or less protractile, with a single row of premaxillary teeth.

*Hemiodus unimaculatus:* **Ecuador:** MECN-DP 6235, 1, 180 mm SL.

Fig. 6B, (Table 2)

**Identification**. Very elongated body. Its silver coloration is slightly darker on the back, with a rounded spot in the middle of the body. The lower lobe of the caudal fin has a dark band above it. The lower terminal mouth is more or less protractile, with a single row of premaxillary teeth.

*Abramites hypselonotus:* **Ecuador:** MECN-DP 6357, 1, 150 mm SL.

Fig. 6C, (Table 2)

**Identification**. The body is deep, with a small head and a forward-positioned mouth. It is light beige in color with dark vertical bands running along the body. The vertical band located in the middle of the body is darker and extends to the base of the dorsal fin rays. The caudal fin is transparent. The adipose fin has a lighter, rounded spot in the center and on the edges.

*Leporinus agassizii:* **Ecuador:** MECN-DP 6373, 1, 210 mm SL; MECN-DP 6399, 1, 201 mm SL; MECN-DP 6400, 1, 170 mm SL.

Fig. 6D, (Table 2)

**Identification**. Medium-sized fish. Laterally compressed body. Mouth positioned terminally with cusped teeth. The species is distinguished by a longitudinal band that begins at the level of the dorsal fin. It has 38 to 40 scales on the lateral line.

*Leporinus friderici:* **Ecuador:** MECN-DP 6232, 1, 208 mm SL; MECN-DP 6254, 1, 139 mm SL; MECN-DP 6363, 1, 175 mm SL.

Fig. 6E, (Table 2)

**Identification**. Elongated body. It has transverse dorsal bands and three oblique spots along the lateral line. Its mouth is small and non-protractile, with no more than four incisors. The fins are slightly yellowish in color.

*Leporinus pearsoni:* **Ecuador:** MECN-DP 6358, 1, 240 mm SL; MECN-DP 6408, 1, 210 mm SL.

Fig. 6F, (Table 2)

**Identification**. The body is spindle-shaped, light brown on the dorsal half, and pale yellow to whitish on the ventral half. It has six striking stripes running from the operculum to the caudal peduncle. The bands under the dorsal fin are usually V-shaped. Hyaline fins.

*Leporinus* sp.: **Ecuador:** MECN-DP 6421, 1, 250 mm SL.

Fig. 6G, (Table 2)

**Identification.** The body is cylindrical and gray, with a pale belly that is slightly yellowish toward the head. No pattern of spots or dots was evident on the dorsal region of the body. All fins are more or less hyaline. The mouth is subterminal.

*Schizodon fasciatus:* **Ecuador:** MECN-DP 6360, 2, 189–192 mm SL; MECN-DP 6361, 1, 250 mm SL; MECN-DP 6362, 1, 230 mm SL.

Fig. 6H, (Table 2)

**Identification**. A medium-sized fish with a laterally compressed, moderately elongated body. This species is distinguished by its multicuspid teeth, which together form a crenulated edge. It has 8 + 1 + 13 gill arches and four black transverse bands on its body, with a caudal spot separate from these bands.

*Curimatella aspera:* **Ecuador:** MECN-DP 6385, 2, 145–155 mm SL.

Fig. 6I, (Table 2)

**Identification**. Fish with a moderately elongated and compressed body. Silvery in color. Their mouths are toothless. They are characterized by their scales being rough to the touch.

*Curimatella meyeri:* **Ecuador:** MECN-DP 6379, 1, 140 mm SL.

Fig. 6J, (Table 2)

**Identification**. A medium-sized fish. It is distinguished by its moderately deep body and scales covering most of the caudal fin lobes. The species has 35 to 39 scales on the lateral line and 6 to 7 transverse scales from the lateral line to the dorsal fin origin. Its coloration is silvery, with hyaline to yellowish fins.

*Potamorhina latior*: **Ecuador:** MECN-DP 6386, 1, 210 mm SL; MECN-DP 6387, 1, 200 mm SL.

Fig. 6K, (Table 2)

**Identification**. A medium-sized fish, distinguished by its deep body and eleven to sixteen unbranched anal fin rays. This species has a ventral keel from the postopercular region to the anal fin base. It has eighty-three to fifty-five scales on the lateral line. Its color is silvery. The fins are hyaline, but the caudal fin has a dark band at the outer end. The scales are very small.

*Psectrogaster amazonica:* **Ecuador:** MECN-DP 6389, 1, 155 mm SL; MECN-DP 6390, 1, 152 mm SL.

Fig. 6L, (Table 2)

**Identification**. A medium-sized fish with a moderately deep body. It has a tripartite lateral snout, a serrated postventral keel, and a dark patch at the base of the median rays of the caudal fin. Head somewhat conical, with large eyes. The body color is silvery, with hyaline fins.

*Psectrogaster* sp.: **Ecuador:** MECN-DP 6422, 1, 135 mm SL; MECN-DP 6423, 1, 152 mm SL.

(Table 2)

**Identification**. Fusiform body with a conical head. The mouth is subterminal, the jaws toothless, and scales are only present at the base of the caudal rays. Silvery in color. The fins are hyaline.

*Steindachnerina guenteri:* **Ecuador:** MECN-DP 6259, 3, 117 mm SL.

Fig. 6M, (Table 2)

**Identification**. Fusiform body with a conical head. Silvery in color with a light brown dorsal surface and body; caudal peduncle with a horizontally elongated black spot starting at the end of the adipose fin and extending along the caudal mid-rays to half its length; dorsal fin with a conspicuous, shapeless black spot located halfway along the dorsal mid-rays. Fins are hyaline except for the caudal, which is yellowish.

*Prochilodus nigricans*: **Ecuador:** MECN-DP 6351, 1, 270 mm SL.

Fig. 6N, (Table 2)

**Identification**. A medium-sized fish with a laterally compressed and fusiform body. It is distinguished by its silvery coloration and hyaline fins, combined with family characteristics such as ever (Tab., disc-shaped lips with tiny, spoon-shaped teeth anchored to very fleshy lips. The caudal fin has dark spots; it also has 44 to 49 scales on the lateral line.

*Parodon pongoensis:* **Ecuador:** MECN-DP 6294, 1, 64 mm SL.

Fig. 7A, (Table 2)

**Figure 7:**
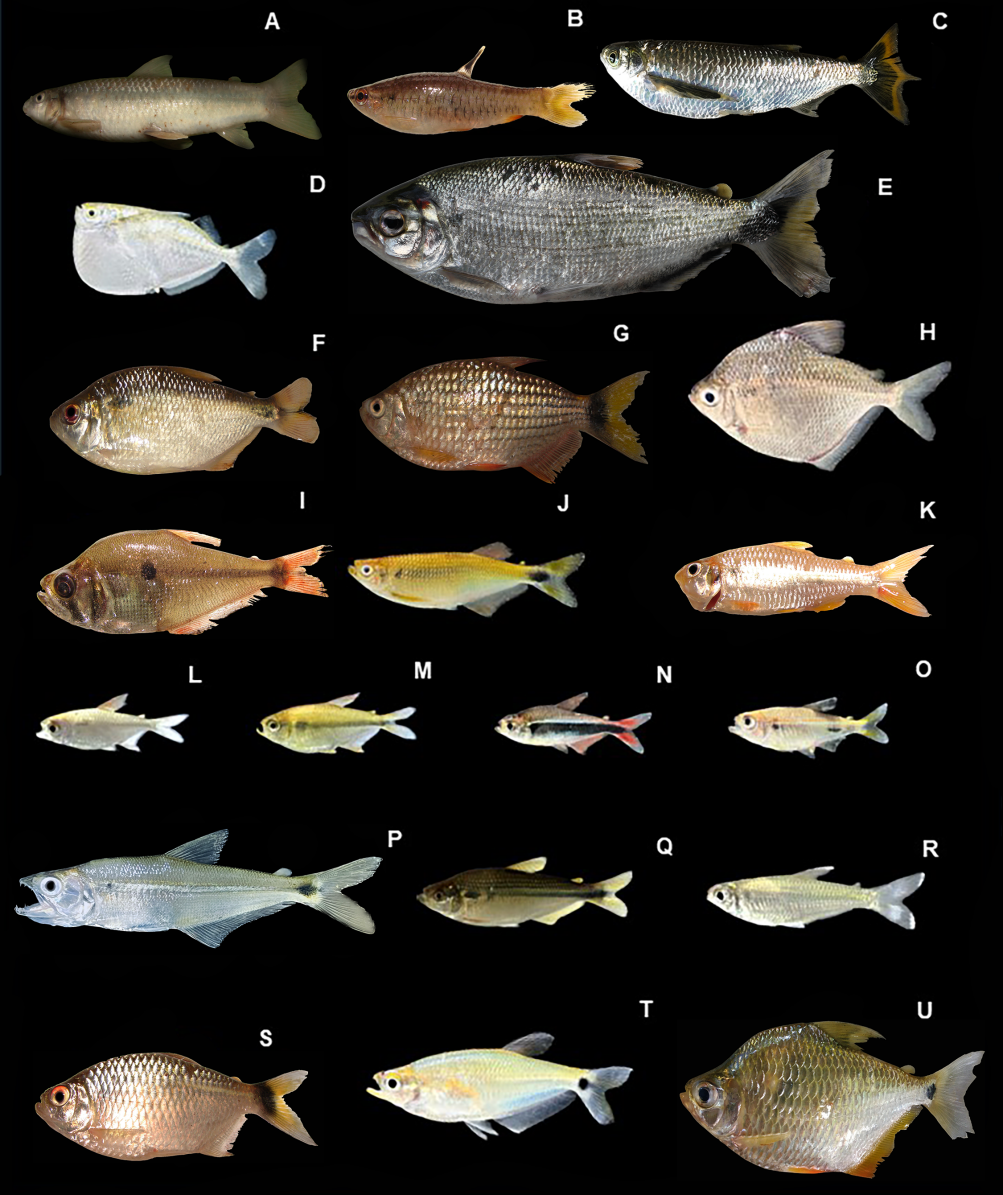
Representative Characiformes of the Conambo River. Shiwiar and Kichwa names available are included as depicted in the dataset. Shi: name in Shiwiar, Ki: name in Kichwa. (A). *Parodon pongoensis* (Shi: Achu Tsarur). (B) *Pyrrhulina semifasciata* (Shi: Kuum). (C) *Triportheus angulatus* (Shi: Saapap). (D) *Thoracocharax stellatus* (Shi: Mitiap). (E) *Brycon melanopterus* (Shi: Singuiatam, Ki: Shangatima). (F) *Astyanax bimaculatus* (Ki: Chul). (G) *Bario steindachneri*. (H) *Brachychalcinus nummus* (Shi: Tundachimi). (I) *Charax tectifer* (Shi: Mapshum, Ki: Galamato). (J) *Chrysobrycon hesperus* (Shi: Wintsa Trarur). (K) *Creagrutus barrigai* (Shi: Wapuk, Ki: Chinlus). (L) *Hemigrammus* aff. *ocellifer*. (M) *Hyphessobrycon agulha* (Shi: Wincha Tsarur). (N) *Hyphessobrycon loretoensis* (Shi: Chumakii nuwari). (O) *Hyphessobrycon* sp. (P) *Galeocharax gulo* (Shi: Kushim). (Q) *Moenkhausia comma* (Shi: Wintsa Trarur). (R) *Moenkhausia dichroura* (Shi: Wapuk, Ki: Cholo). (S) *Moenkhausia oligolepis* (Shi:Yantsar). (T) *Paragoniates alburnus* (Shi: Taangas). (U) *Tetragonopterus argenteus* (Shi: Churuind wancha, Ki: Casapa).

**Identification**. It is characterized by a wide, dark midlateral stripe above the lateral line, extending from the snout to the tip of the median rays of the caudal fin. The body coloration above the lateral stripe is darker than the lower half, which is whitish. All fins are hyaline, the caudal fin is bilobed, adipose, and the anal fin is short.

*Pyrrhulina eleanorae:* **Ecuador:** MECN-DP 6345, 2, 67–68 mm SL; MECN-DP 6348, 2, 40–51 mm SL.

(Table 2)

**Identification**. A small fish. It has two complete rows of teeth in the upper jaw, a mouth positioned terminally. The roof of the head lacks fontanelles. It has a black line extending from the eye to the second or third scale. It has eleven predorsal scales.

*Pyrrhulina semifasciata:* **Ecuador:** MECN-DP 6293, 1, 52 mm SL; MECN-DP 6327, 5, 30–40 mm SL; MECN-DP 6328, 1, 40 mm SL; MECN-DP 6346, 1, 219 mm SL.

Fig. 7B, (Table 2)

**Identification**. Slightly elongated body; characterized by a silvery to grayish coloration with a longitudinal band extending from the tip of the snout to just before the middle of the body. The pectoral fin is hyaline, and the ventral and anal fins are orange in color on the distal part. The anterior ventral region of the head and upper mouth have conical teeth, one series on the mandible and two series on the premaxilla.

*Triportheus angulatus*: **Ecuador:** MECN-DP 6234, 1, 160 mm SL; MECN-DP 6378, 1, 212 mm SL; MECN-DP 6401, 2, 145–230 mm SL.

Fig. 7C, (Table 2)

**Identification**. Elongated, compressed body with a ventral keel. Deep silver in color, with yellowish fins with slightly faint edges. Large scales. Well-developed pectoral fins. The median rays of the caudal fin protrude like a filament.

*Triportheus sp.:* **Ecuador:** MECN-DP 6307, 1, 60.7 mm SL.

(Table 2)

**Identification.** The body is strongly laterally compressed and relatively deep, with a straight or slightly concave ventral profile that includes a well-developed abdominal keel bordered by keeled scales. The head is short with large eyes, and the mouth is superior, adapted for surface feeding. The teeth are small, conical or slightly tricuspid, arranged in one or two rows. The lateral line is complete and conspicuous. Its overall color is silvery with metallic reflections. The soft fin rays have an adipose fin and an elongated anal fin. The caudal fin is deeply forked.

*Thoracocharax stellatus*: **Ecuador:** MECN-DP 6253, 2, 67–69 mm SL.

Fig. 7D, (Table 2)

**Identification**. A small, silvery fish with hyaline fins and a black spot at the base of the dorsal fin. It has nineteen to twenty-two longitudinal scales and also has an adipose fin. The ventral keel is very pronounced. Body very laterally compressed. Well developed pectoral fins. Short head. Mouth positioned dorsally.

*Brycon melanopterus:* **Ecuador:** MECN-DP 6233, 1, 205 mm SL; MECN-DP 6359, 1, 240 mm SL.

Fig. 7E, (Table 2)

**Identification**. A robust fish, silvery in color, with a darker back. It has a diagonal dark spot that extends from the base of the pelvic fins to the distal part of the upper caudal lobe.

*Astyanax bimaculatus:* **Ecuador:** MECN-DP 6272, 1, 49 mm SL; MECN-DP 6299, 1, 74.3 mm SL; MECN-DP 6380, 1, 89 mm SL.

Fig. 7F, (Table 2)

**Identification**. A medium-sized species with an oval, silvery body. The genus has two rows of premaxillary teeth, five teeth in the inner premaxillary series, a complete lateral line, and the presence of an adipose fin. The species is characterized by yellow to orange fins, a horizontally elongated, rhomboid-shaped peduncular spot, and a circular, also horizontally elongated humeral spot. The body is tall and laterally compressed.

*Astyanax* sp.: **Ecuador:** MECN-DP 6337, 1, 27 mm SL.

(Table 2)

**Identification**. Medium-sized species. Body silvery with a slightly darkened back. No conspicuous humeral spot. Premaxilla with two rows of teeth, the outer one with four and the inner one with five; dentary with four teeth; maxilla without teeth.

*Bario steindachneri:* **Ecuador:** MECN-DP 6311, 1, 60 mm SL.

Fig. 7G, (Table 2)

**Identification**. A small species with a deep, compressed body and a slightly keeled pre-thoracic area. It is yellowish in color with a diffuse humeral spot, a very conspicuous caudal spot, and a series of longitudinal stripes on the sides between the scale rows.

*Brachychalcinus nummus*: **Ecuador:** MECN-DP 6250, 1, 88 mm SL.

Fig. 7H, (Table 2)

**Identification**. Small species. Deep body, rounded to rhomboidal, highly compressed laterally, with a vertically elongated humeral spot; body silvery. It has a predorsal spine and a preanal spine, with eight to twelve scales on the lateral line. Head short, with large eyes.

*Charax tectifer*: **Ecuador:** MECN-DP 6317, 4, 40–60 mm SL; MECN-DP 6329, 1, 38 mm SL.

Fig. 7I, (Table 2)

**Identification**. A small, laterally compressed fish. It is distinguished by a predorsal hump and a pronounced groove on the posteroventral margin of the cleithrum. Strong jaws, with well developed caniniform teeth, and moderately elongated snout. The species is distinguished by the reddish coloration of its fins, with a black outer edge, and by the presence of a rounded lateral midspot.

*Chrysobrycon hesperus:* **Ecuador:** MECN-DP 6315, 1, 55 mm SL.

Fig. 7J, (Table 2)

**Identification.** Small species. Body slightly elongated and compressed. The dorsal fin is located in the last third of the body. It is silvery in color. All fins are hyaline. Upper mouth with tricuspid teeth.

*Creagrutus barrigai*: **Ecuador:** MECN-DP 6291, 1, 89 mm SL.

Fig. 7K, (Table 2)

**Identification**. Body spots, vertically elongated humeral mark, laterally compressed body. Absence of a series of dark mediolateral scales. Two or three maxillary teeth, five teeth on each dentary, 36–38 vertebrae, 13–15 branched anal fin rays, 8–10 predorsal scales, four scale rows between the lateral line and the dorsal fin origin, six–eight gill rakers on the upper branch of the first gill arch, the third infraorbital well developed in contact with the horizontal branch of the preoperculum.

*Hemigrammus* aff. *ocellifer*: **Ecuador:** MECN-DP 6291, 1, 48 mm SL.

Fig. 7L, (Table 2)

**Identification**. A tiny, spindle-shaped species. The golden line runs along the lateral line, becoming more pronounced at the top of the caudal peduncle. The dorsal, pectoral, and pelvic fins, and half of the anal fin rays, are hyaline.

*Hemmigrammus ocellifer*: **Ecuador:** MECN-DP 6344, 2, 28–30 mm SL.

(Table 2)

**Identification**. A tiny species. It has two very conspicuous dark humeral spots, followed by a thin line extending to the caudal peduncle, where a rhomboid spot is evident that reaches the caudal midrays. It has a small iridescent red band along the midline of the caudal peduncle. The caudal, pelvic, and pectoral fins are yellowish. The caudal fin is relatively red.

*Hyphessobrycon agulha*: **Ecuador:** MECN-DP 6308, 2, 25–26 mm SL; MECN-DP 6338, 6, 20–30 mm SL; MECN-DP 6339, 2, 25 mm SL.

Fig. 7M, (Table 2)

**Identification**. Short and relatively tall body. Head with a large eye, iris with a deep red upper edge. The body is silvery; the predorsal region is reddish-brown, and the belly is silvery; from the posterior edge of the gill to the tips of the central rays of the caudal fin, there is a longitudinal black band. Hylaine fins.

*Hyphessobrycon loretoensis:* **Ecuador:** MECN-DP 6263, 1, 23.5 mm SL.

Fig. 7N, (Table 2)

**Identification**. Relatively robust, fusiform body. It has a golden band along the lateral line. Below this, between the operculum and the caudal peduncle, it has a wide black band that continues toward the underside of the body. The entire caudal fin is bright brick red. The dorsal, pectoral, ventral, and anal fins are light red.

*Hyphessobrycon* sp.: **Ecuador:** MECN-DP 6304, 3, 27.5–30 mm SL.

Fig. 7O, (Table 2)

**Identification**. Silvery body with a dark spot on the caudal fin. The iris has a reddish rim. The dorsal, anal, and adipose fins and caudal fin lobes are orange.

*Galeocharax gulo*.: **Ecuador:** MECN-DP 6282, 1, 91 mm SL.

Fig. 7P, (Table 2)

**Identification**. Fish with an elongated body, a long mouth, and canine-shaped teeth. Silvery yellow in color with a vertically elongated humeral spot followed by a silver band extending to the caudal peduncle.

*Knodus gamma:* **Ecuador:** MECN-DP 6287, 1, 51 mm SL.

(Table 2)

**Identification**. A very small fish. Fusiform body, laterally compressed. It is distinguished by the scales on its caudal fin, a vertically elongated humeral spot, and two sets of premaxillary teeth. Its body color is silver. The fins have an orange coloration. In this species, the dorsal fin is located in the anterior half of the body. It also has 23 to 30 branched rays on the anal fin and 35 to 39 scales on the lateral line.

*Knodus megalops:* **Ecuador:** MECN-DP 6274, 2, 34–43 mm SL.

(Table 2)

**Identification**. A small, elongated species. Its body is semitransparent and silvery. It has a complete lateral line. Its fins are hyaline.

*Moenkhausia comma:* **Ecuador:** MECN-DP 6298, 1, 47.6 mm SL.

Fig. 7Q, (Table 2)

**Identification**. A species with a very tall, compressed body. Silvery in color, with a horizontally elongated humeral patch, wider and rounded at the posterior end, resembling a comma. Fins are somewhat reddish. The caudal fin is scaled on at least one-quarter of the lobes.

*Moenkhausia dichroura:* **Ecuador:** MECN-DP 6324, 5, 32–42 mm SL.

Fig. 7R, (Table 2)

**Identification.** The body is fusiform, moderately laterally compressed, with a short head and large eyes, and tricuspid premaxillary teeth arranged in two rows. It has a complete lateral line. The mouth is terminal. Fins have soft rays. The coloration is generally silvery with golden reflections on the back, with a prominent dark or silvery longitudinal band extending from the posterior edge of the operculum to the base of the caudal peduncle. The caudal lobes have distal spots that do not cover the tips. The other fins are hyaline.

*Moenkhausia oligolepis:* **Ecuador:** MECN-DP 6295, 1, 60.9 mm SL; MECN-DP 6313, 4, 64–69 mm SL.

Fig. 7S, (Table 2)

**Identification.** The body is fusiform, laterally compressed, and small to medium in size. The head is proportional, with large eyes and a non-protractile terminal mouth. The premaxillary teeth are tricuspid and arranged in two rows. The lateral line is complete. The body is silvery with greenish or golden hues on the back, with a faint humeral spot and a conspicuous black spot on the caudal peduncle. The fins are hyaline, although they may have slightly orange or reddish hues on the anal and caudal fins. The caudal fin is forked, with symmetrical lobes.

*Paragoniates alburnus:* **Ecuador:** MECN-DP 6243, 1, 59 mm SL; MECN-DP 6281, 1, 52 mm SL.

Fig. 7T, (Table 2)

**Identification.** The body is elongated and fusiform, moderately compressed laterally. It has a small head with large eyes and a terminal mouth, slightly upward-facing, with tricuspid teeth arranged in bands on the premaxilla and dentary. The lateral line is incomplete. The general coloration is silvery, with a darker back and bluish or greenish reflections; a dark longitudinal band extends from the operculum to the base of the caudal fin. The fins are mostly hyaline, although in live specimens, pink or orange tones can be observed in the anal and caudal fins. The dorsal fin is located behind the origin of the anal fin; the adipose fin is present. The caudal fin is forked and symmetrical.

*Tetragonopterus argenteus:* **Ecuador:** MECN-DP 6354, 1, 101 mm SL; MECN-DP 6355, 1, 90 mm SL.

Fig. 7U, (Table 2)

**Identification.** The body is deep, laterally compressed, and rhomboid in shape, with a convex dorsal profile. The head is short, with large eyes and a terminal mouth with pentacuspid teeth arranged in two bands on the premaxilla. The lateral line is complete. The body is bright silver in color, with bluish or greenish reflections on the back and a lighter hue on the belly. The fins have two vertically elongated humeral spots and a faint longitudinal band and a diffuse dark spot on the caudal peduncle. The fins are generally bright orange, and the caudal fin is forked with symmetrical lobes.

*Cetopsis coecutiens:* **Ecuador:** MECN-DP 6273, 1, 50.1 mm SL; MECN-DP 6356, 1, 220 mm SL; MECN-DP 6406, 1, 170 mm SL.

Fig. 8A, (Table 2)

**Figure 8:**
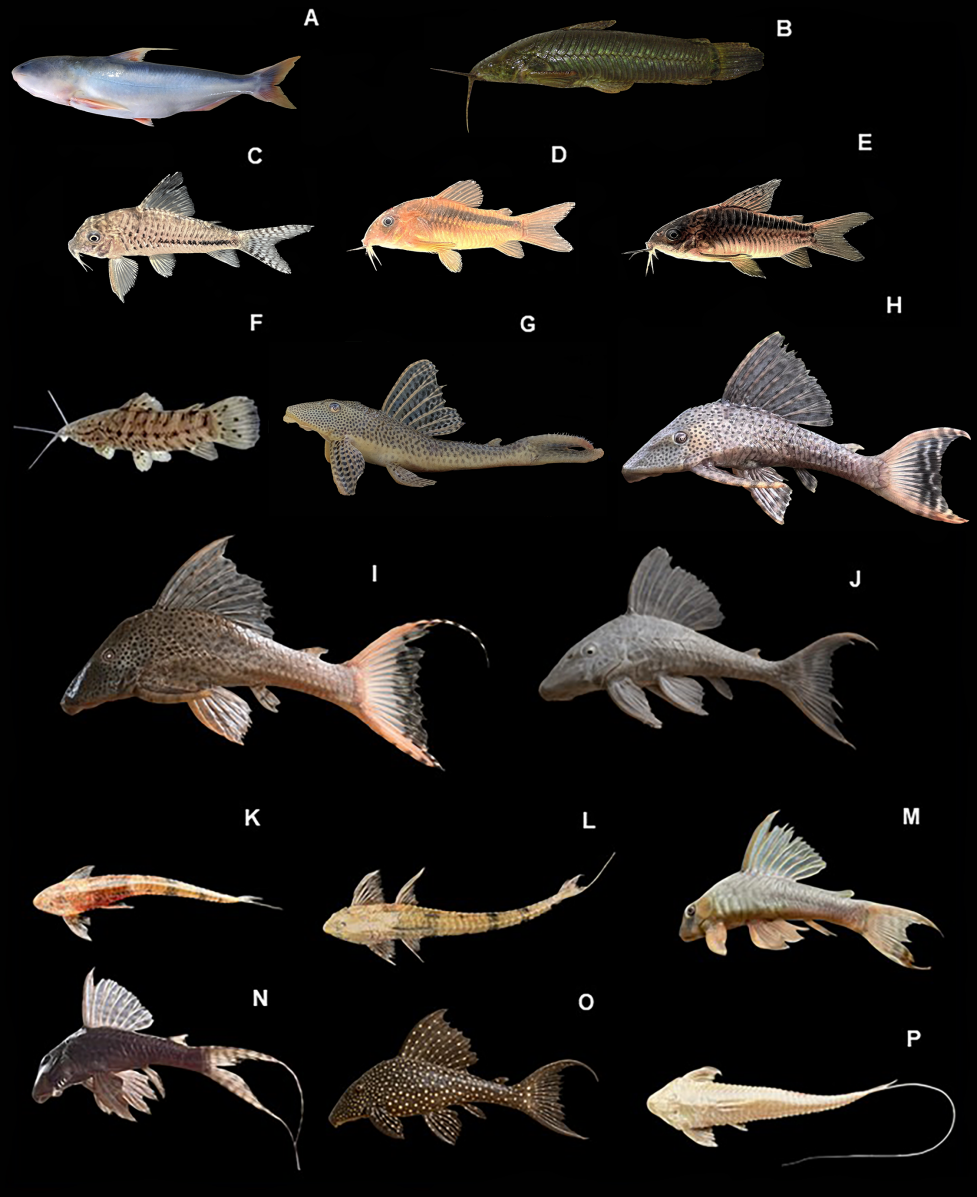
Representative Siluriformes of the Conambo River. Shiwiar and Kichwa names available are included as depicted in the dataset. Shi: name in Shiwiar, Ki: name in Kichwa. (A) *Cetopsis coecutiens*. (B) *Callichthys callichthys*. (C) *Corydoras aeneus* (Shi: Yuyui). (D) *Corydoras rabauti* (Shi: Sharamyuyui). (E) *Corydoras zygata* (Shi: Kusum). (F) *Megalechis thoracata*. (G) *Aphanotorulus unicolor* (Shi: Numipuutu, Ki: Asnak shiyu). (H) and (I) *Hypostomus cochliodon*. (J) *Hypostomus niceforoi*. (K) *Loricaria simillima*. (L) *Loricariichthys* sp. (Shi: Singuiag). (M) *Panaqolus dentex*. (N) *Panaqolus nocturnus*. (O) *Panaqolus pantostiktos*. (P) *Pseudohemiodon* sp.

**Identification.** The body is elongated, laterally compressed, and medium to large in size. The head is robust and depressed, with small, laterally located eyes and a broad, terminal to subterminal mouth, with conical and curved teeth arranged in multiple irregular rows on both jaws. It has a pair of maxillary barbels and two pairs of mental barbels. Its body coloration is gray to dark brown on the back and flanks, lighter ventrally, and without conspicuous patterns. The fins are hyaline or slightly dark; the dorsal fin has a single hard ray followed by soft rays, the anal fin is elongated, and the caudal fin is deeply forked.

*Vandellia cirrhosa:* **Ecuador:** MECN-DP 6267, 1, 56 mm SL.

(Table 2)

**Identification.** The body is extremely elongated, slender, and translucent, with a needle-like shape. It has a small, depressed head with tiny eyes and an upper mouth surrounded by three pairs of short barbels. It has no pelvic fins or swim bladder, and its dorsal and anal fins are reduced, with few rays. The caudal fin is small and rounded. The overall body coloration is whitish or pinkish in life.

*Callichthys callichthys:* **Ecuador:** MECN-DP 6381, 2, 100 mm SL.

Fig. 8B, (Table 2)

**Identification.** The body is elongated, somewhat laterally compressed, with a flattened ventral region. Its head is broad and flattened, with medium-sized eyes and a subterminal mouth surrounded by two pairs of sensory barbels. It has a double row of bony plates on each side of the body, from the head to the base of the caudal fin. Its coloration is olive-brown to grayish dorsally, with lighter shades ventrally and irregular spots on the flanks; the fins are hyaline or lightly pigmented. The dorsal fin has a spiny ray followed by soft rays; the pectoral fin also has a strong, serrated spine. The caudal fin is rounded or slightly emarginate.

*Corydoras acutus:* **Ecuador:** MECN-DP 6244, 1, 53 mm SL; MECN-DP 6292, 2, 39–50 mm SL. (Table 2)

**Identification.** The body is robust, elongated, and slightly laterally compressed. It has a broad head with a gently curved dorsal profile, large eyes, and a downward-facing subterminal mouth surrounded by three pairs of sensory barbels. Its body is covered by two rows of bony plates on each side. The overall coloration is silvery-gray to light brown, with a dark band extending longitudinally from the snout to the base of the caudal fin. The fins are transparent or lightly pigmented; the dorsal fin has a spiny ray followed by soft fins, and the caudal fin is truncated or slightly emarginate.

*Corydoras aeneus:* **Ecuador:** MECN-DP 6303, 1, 41.5 mm SL.

Fig. 8C, (Table 2)

**Identification.** Ventrally compressed, elongated, and somewhat fusiform body. The head is broad, with large eyes and a lower mouth surrounded by two pairs of sensory barbels. The body is covered with rows of lateral bony plates. The dorsal fin has a serrated hard ray followed by soft rays; the pectoral fins also have serrated spines. The caudal fin is truncated or slightly forked. The body is bronze-brown or metallic-green, darker on the back and lighter on the belly. It has a distinct dark longitudinal band from the snout to the caudal peduncle. The fins are hyaline or slightly orange, without vertical bands.

*Corydoras rabauti:* **Ecuador:** MECN-DP 6240, 1, 30.2 mm SL.

Fig. 8D, (Table 2)

**Identification.** The body is ventrally compressed, elongated, and slightly fusiform. The head is large and rounded, with prominent eyes and a lower-positioned mouth with two pairs of short barbels. The body is covered with rows of lateral bony plates. The dorsal fin has one hard ray and eight soft rays; the spine is short and the rays are longer and branched. The anal fin has one hard ray and six soft rays. The pectoral fins extend beyond the origin of the pelvic fins; the latter almost reach the anal fin. The caudal fin is truncated or slightly forked. The overall coloration is light brown or coppery, with a metallic blue band running from the posterior margin of the operculum to the caudal peduncle, with a short vertical stripe on the peduncle. An additional blue stripe is located below the eye. The pelvic and dorsal fins are dark orange; the rest of the fins are light orange, except for the caudal fin, which is hyaline.

*Corydoras zygata:* **Ecuador:** MECN-DP 6278, 4, 37.5–43.3 mm SL; MECN-DP 6320, 2, 30–35 mm SL.

Fig. 8E, (Table 2)

**Identification.** The body is slightly laterally compressed and elongated. The head is rounded with large eyes and a ventrally positioned mouth, equipped with two pairs of short, mobile barbels. The dorsal fin has a serrated hard ray followed by soft rays; the pectoral fins have serrated spines. The caudal fin is forked. The overall body coloration is light brown with a distinctive black longitudinal band extending from the snout to the base of the caudal fin. Rounded spots may be present on the caudal peduncle. The fins are hyaline, with possible orange or pinkish tinges, and without visible bands.

*Megalechis thoracata:* **Ecuador:** MECN-DP 6238, 1, 56.6 mm SL; MECN-DP 6285, 1, 75 mm SL; MECN-DP 6300, 3, 24.3–39.4 mm SL; MECN-DP 6309, 4, 55–80 mm SL; MECN-DP 6310, 1, 74 mm SL; MECN-DP 6347, 1, 50 mm SL.

Fig. 8F, (Table 2)

**Identification.** Robust body, elongated and slightly laterally compressed. Head broad, dorsoventrally flattened, with a subterminal, extensible mouth and two pairs of barbels (one maxillary and one mental). Dorsal fin with a serrated hard ray followed by soft rays; pectoral fin with a thick, strongly serrated spine. Caudal fin usually truncated or slightly emarginate. Body coloration dark brown to grayish, with scattered darker spots on the flanks and back; ventral region lighter. May have a faint longitudinal band. Fins hyaline with grayish or orange tints and irregular dark spots, especially on the caudal fin.

*Ancistrus malacops:* **Ecuador:** MECN-DP 6286, 2, 75–79 mm SL.

(Table 2)

**Identification.** Body dorsoventrally depressed, elongated and flattened, especially in the cephalic and anterior regions of the trunk. Ventrally positioned, sucker-shaped mouth. Premaxillary teeth incisor, asymmetrically bifid, arranged in a single row, with the medial cusp longer and wider than the lateral one. Presence of rosette-shaped, mobile, well-developed odontodes on the cheeks, between 10 and 12. Dorsal fin with a hard ray followed by soft rays; caudal fin typically truncated or slightly emarginate. General coloration dark brown to blackish, with light spots or beige mottling irregularly distributed over the body and fins, often forming cryptic patterns. Pectoral, ventral and dorsal fins with light spots or faint bands; caudal fin may have dark spots or bands.

*Ancistrus shuar:* **Ecuador:** MECN-DP 6255, 1, 104 mm SL.

(Table 2)

**Identification.** Body robust and depressed anteriorly, slightly compressed towards the caudal peduncle; head broad and depressed. Mouth extensible, subterminal, oval or rounded, with a narrow upper lip covering the premaxilla; the hemimandibles are straight or form an open V. The premaxillary and dentary teeth are incisor-type, asymmetrically bifid, with the medial cusp longer and wider than the lateral, arranged in a single row, and are numerous and tiny. The cheeks have well-developed, rosette-shaped mobile odontodes that can almost reach the origin of the pectoral fin. Dorsal fin with a hard ray followed by soft rays; caudal fin truncated or slightly emarginate. Overall coloration is dark brown with pale mottling all over the body and fins; a cryptic pattern that favors camouflage on rocky bottoms. Fins with pale spots or faint bands; caudal fin with dark edges and light areas towards the center.

*Aphanotorulus unicolor:* **Ecuador:** MECN-DP 6376, 2, 165–170 mm SL; MECN-DP 6382, 1, 160 mm SL.

Fig. 8G, (Table 2)

**Identification.** Body dorsoventrally depressed, elongated, and robust, with bony plates covering most of the body except the ventral region of the abdomen. The mouth is ventrally positioned, sucker-shaped, with well-developed lips. The premaxillary and dentary teeth are conical and arranged in a single row. The dorsal fin is large, with a hard ray followed by soft rays; the caudal fin is strongly forked. The adipose fin has a membrane that extends to the end of the fourth adipose plate. The body is light brown with a white ventral region; dark rounded spots are observed on the dorsal and lateral surfaces in longitudinal rows. The spots on the caudal fin form vertical bars when compressed, and on the dorsal fin, they are aligned before each ray.

*Farlowella knerii:* **Ecuador:** MECN-DP 6251, 1, 141 mm SL.

(Table 2)

**Identification.** Extremely elongated and slender body, strongly compressed dorsoventrally in the cephalic region. Head elongated, tube-shaped, with a projecting snout; mouth ventrally positioned, suction-cup-shaped. The upper and lower jaws are provided with conical teeth arranged in a single row. Dorsal fin small, with one hard ray and several soft rays; caudal fin truncated or slightly emarginate. General coloration light brown to greenish, distinguished by the presence of dark dorsolateral stripes that begin at the base of the snout, pass through the eye, and fade before the dorsal fin. Fins hyaline with yellowish-brown tones. A brown spot extends from the caudal peduncle to the upper lobe of the caudal fin.

*Hypostomus cochliodon:* **Ecuador:** MECN-DP 6395, 7, 159–170 mm SL; MECN-DP 6396, 6, 150–160 mm SL; MECN-DP 6413, 1, 155 mm SL; MECN-DP 6414, 1, 149 mm SL.

Figs. 8H–8I, (Table 2)

**Identification.** The body is robust, slightly depressed dorsally and flattened ventrally, with an elongated, fusiform shape. The head is broad and flattened, with a ventrally positioned, sucker-shaped mouth, and short, broad, strongly spatulate premaxillary and dentary teeth arranged in a single row. Odontodes are present, arranged in a rosette along the body, and are more developed in reproductive males, especially on the margins of the body and fins. The dorsal fin has one hard ray and seven soft rays, tall and sustained; the pectoral fins are robust with spiny rays. The caudal fin is generally truncated or slightly emarginate. The overall coloration is dark brown or grayish, with lighter spots or vermiculations irregularly distributed over the body and fins; some individuals are uniformly colored.

*Hypostomus niceforoi:* **Ecuador:** MECN-DP 6366, 3, 131–167 mm SL; MECN-DP 6405, 1, 15 mm SL.

Fig. 8J, (Table 2)

**Identification.** The body is robust, slightly depressed dorsoventrally, elongated, and fusiform. The head is broad, flat, and has a straight dorsal profile. Odontodes are present, distributed on the head, trunk, and fins, and are more developed in reproductive males. The mouth is ventrally positioned, with jaws provided with fine, long, bilobed teeth, one of which has a more developed apex and a curved tip. The dorsal fin has one hard ray and seven soft rays; the pectoral fins have robust, spiny rays. The caudal fin is truncated or slightly emarginate, with a slightly shorter upper lobe. The background color is greenish-brown to brown, with numerous rounded or elongated dark spots covering the body and fins, forming a characteristic reticulated pattern. The fins have irregular bands or dark dots.

*Lasiancistrus heteracanthus:* **Ecuador:** MECN-DP 6246, 1, 60 mm SL.

(Table 2)

**Identification.** Body dorsoventrally depressed, elongated, and fusiform. Head broad, flattened, with rounded margins; mouth ventrally positioned, sucker-shaped. Upper lip with small, rounded papillae, while the lower lip has medium-sized papillae anteriorly and smaller posteriorly. The teeth are elongated, with narrow, thin cusps and distinctly long stalks. Odontodes are present on the head and body, including rosette-shaped structures on the margins of the operculum and along the flanks; reproductive males have elongated odontodes on the body margins and on the pectoral fins. Dorsal fin with one hard ray and seven soft rays; pectoral fins stout, with conspicuous spines. Caudal fin truncate or slightly emarginate, the lower lobe longer than the upper. Dark brown to grayish background coloration with scattered light spots on the body and fins; usually with a light band along the base of the dorsal fin.

*Limatulichthys griseus:* **Ecuador:** MECN-DP 6260, 1, 66.8 mm SL.

(Table 2)

**Identification.** Body elongated and dorsoventrally depressed, fusiform in shape. Head broad and flattened, with dorsolateral eyes; ventrally positioned, suction-cup-shaped mouth. Fine odontodes are present on the head and body, especially on the margins of the head and pectoral fins. Dorsal fin with a hard ray and at least seven soft rays; pectoral and pelvic fins broad, adapted for support on the substrate. Caudal fin generally emarginate. Overall coloration grayish or light brown with a uniform or slightly mottled pattern; the belly is lighter.

*Loricaria* cf. *clavipina*: **Ecuador:** MECN-DP 6393, 1, 200 mm SL.

(Table 2)

**Identification.** Body elongated and strongly depressed dorsoventrally, with a flat ventral profile. Head broad, triangular, with dorsolateral eyes. Mouth positioned ventrally, sucker-shaped, upper lip has long tentacles or barbels, and the lower lip is wide, covered with numerous short cirri. The teeth are bilobed, with a small, pointed outer lobe and a more rounded and elongated inner lobe. Small odontodes are distributed along the body, especially concentrated on the head, edges of the operculum and pectoral fins. Dorsal fin with a spiny ray followed by several soft rays; pectoral fins large, with rigid spines and soft rays; caudal fin truncated or slightly emarginate. Overall coloration is light brown to dark brown, with irregular dark spots along the body and fins; belly is lighter. The caudal fin has a transverse black spot and the anal fin is lighter in color.

*Loricaria simillima:* **Ecuador:** MECN-DP 6245, 1, 90 mm SL; MECN-DP 6264, 1, 75.2 mm SL.

Fig. 8K, (Table 2)

**Identification.** Body elongate, dorsoventrally depressed and covered by bony plates, with a somewhat depressed shape towards the caudal peduncle. Head broad, triangular in shape, with dorsolateral eyes. Ventral mouth shaped like a sucker. It has short, dense odontodes, most visible on the head, opercular margins and pectoral fins. Dorsal fin with a spiny ray followed by soft rays; pectoral fins robust, with rigid spines and soft rays; caudal fin truncated or slightly emarginate. Overall coloration light brown to dark brown, with scattered dark spots on the body and fins; ventral region lighter.

*Loricariichthys sp.:* **Ecuador:** MECN-DP 6279, 2, 55.3–123.6 mm SL.

Fig. 8L, (Table 2)

**Identification.** Body elongated and dorsoventrally depressed. Head triangular, broad, and flattened, with small dorsolateral eyes. Ventral mouth shaped like a sucker, with broad lips provided with papillae; short barbels present. Short odontodes on head and pectoral region, more developed in breeding males. Dorsal fin with a spiny ray followed by soft rays; pectoral fins robust with stiff spine; caudal fin typically truncated or slightly emarginate. Light brown to greyish coloration, with diffuse spots or bands on the body and fins, and a paler belly.

*Panaqolus dentex:* **Ecuador:** MECN-DP 6288, 1, 65 mm SL.

Fig. 8M, (Table 2)

**Identification.** The body is robust, laterally compressed, elongated, and fusiform. The head is broad, with a straight and slightly sloping dorsal profile. The ventral mouth has an oral disc ranging from ovoid to rhomboidal, with elongated maxillary barbels. The teeth are unicuspid and spoon-shaped, with a number varying between four and six in the premaxillaries and between five and eight in the dentaries. Straight and curved odontodes are present on the head and fin spines, more developed in breeding males. The dorsal fin has one hard ray and seven soft rays, is tall and has extended membranes; the pectoral fins are robust; the caudal fin is generally emarginate to forked. The overall coloration is dark brown with lighter or yellowish vertical bands along the body and fins, a pattern that may vary according to size and location.

*Panaqolus nocturnus*: **Ecuador:** MECN-DP 6276, 1, 52 mm SL; MECN-DP 6397, 2, 140–150 mm SL; MECN-DP 6398, 4, 130–150 mm SL; MECN-DP 6404, 1, 120 mm SL; MECN-DP; 6415, 1, 131 mm SL; MECN-DP 6416, 1, 115 mm SL.

Fig. 8N, (Table 2)

**Identification.** The body is robust, laterally compressed, and elongated, covered by plates. The head is broad and dorsally flattened. The ventral mouth is sucker-shaped, with well-developed lips and numerous papillae. The cheeks have long, straight odontodes with recurved tips that reach the posterior part of the first lateral plate of the body. The maxillary barbels are elongated, and the teeth are unicuspid and spoon-shaped. The dorsal fin is tall and extended, with one hard ray and seven soft rays; the pectoral and pelvic fins are well-developed; the caudal fin is usually forked. The overall coloration is very dark brown to black, with faint or absent light bands. The fins are dark, with slight light tinges in some specimens.

*Panaqolus pantostiktos:* **Ecuador:** MECN-DP 6402, 1, 143 mm SL.

Fig. 8O, (Table 2)

**Identification.** Body robust, elongated, and slightly laterally compressed. Ventral surface of head and belly with small, irregularly shaped plates, provided with small or very small odontodes. Mouth positioned ventrally, with a rounded to elliptical oral disc; developed and evident maxillary barbels; papillose lips with smooth edges, the lower one being larger and more developed than the upper. Teeth spoon-shaped, unicuspid or with a very small lateral cusp. Presence of curved odontodes on the cheeks and along the margins of the fins, more developed in adult males. Dorsal fin with one hard ray and seven soft rays; well-developed pectoral and pelvic fins; caudal fin truncated or slightly forked. Light brown to creamy background coloration, covered by numerous dark brown spots evenly distributed over the body and fins, a distinctive feature of the species. Fins with the same dotted pattern.

*Peckoltichthys bachi:* **Ecuador:** MECN-DP 6277, 2, 45–56 mm SL; MECN-DP 6375, 1, 90 mm SL; MECN-DP 6384, 1, 109 mm SL.

(Table 2)

**Identification.** Body elongate, robust, and moderately laterally compressed, covered by bony plates without keels on the dorsal and lateral regions. Hypertrophied odontodes are present on the cheeks. Mouth is ventrally positioned, with thin lips; upper lip with small, round papillae; lower lip with medium-sized papillae. Jaws narrow, teeth with small, slightly narrow cusps and long stalks. Dorsal fin with one hard ray and seven soft rays; pectoral and pelvic fins well developed, caudal fin truncated or slightly emarginate. Overall coloration brown to dark brown with darker, irregularly spotted or banded patterns along the body and fins.

*Pseudohemiodon sp.:* **Ecuador:** MECN-DP 6392, 1, 188 mm SL.

Fig. 8P, (Table 2)

**Identification.** The body is depressed, very dorsoventrally flattened, leaf-shaped, and adapted for benthic life. The head is broad and flattened. The ventral mouth is disc-shaped, with broad, papillae-covered lips. The dorsal fin has a hard ray and five to six soft rays; the pectoral fins are broad, fan-shaped, and extend laterally. The caudal fin is truncated or slightly emarginate. The overall coloration is light brown to beige, with mottled or reticulated patterns that facilitate camouflage on sandy bottoms.

*Trachelopterus galeatus* **:** **Ecuador:** MECN-DP 6383, 1, 160 mm SL.

Fig. 9A, (Table 2)

**Figure 9:**
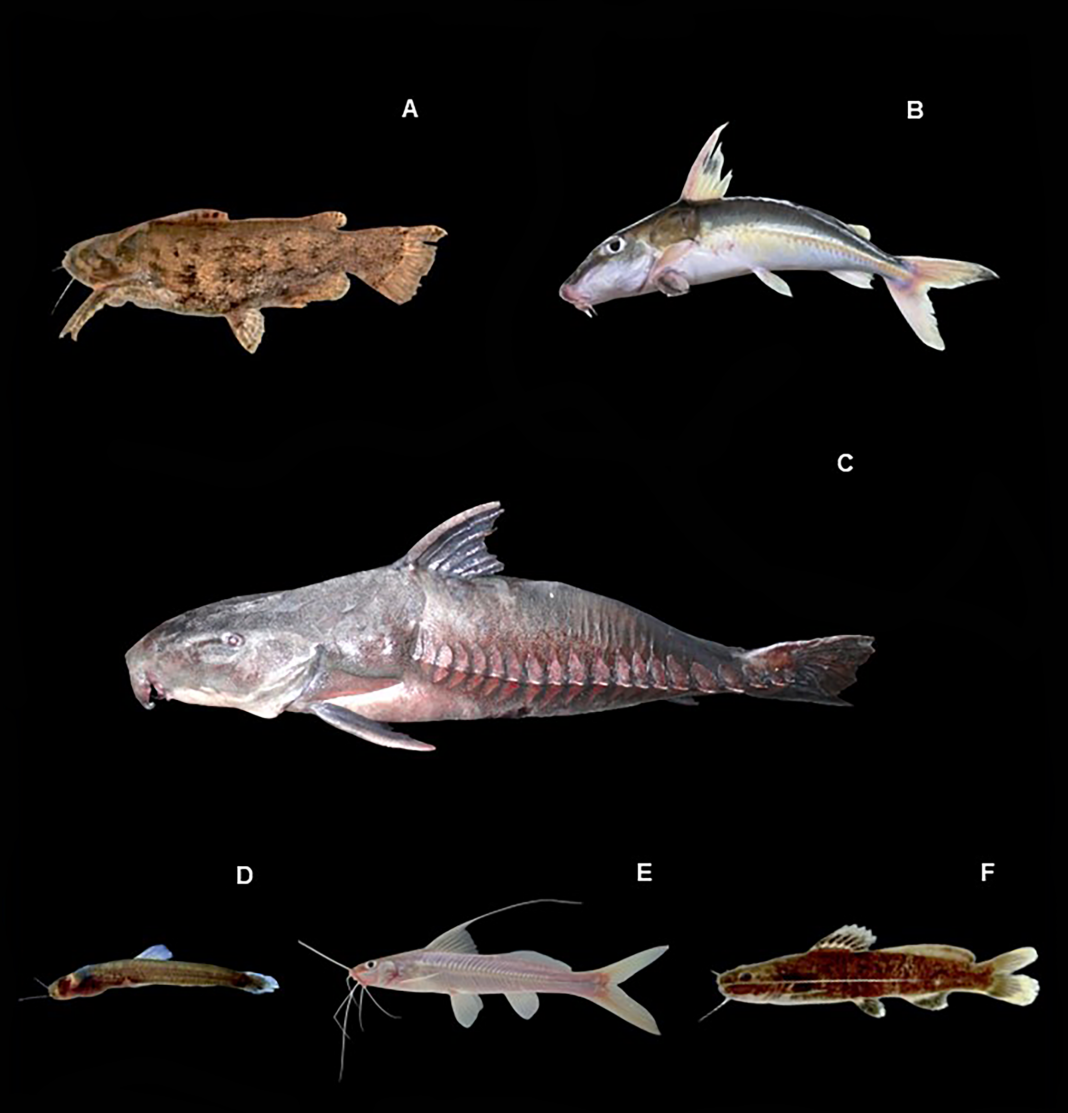
Representative Siluriformes of the Conambo River. Shiwiar and Kichwa names available are included as depicted in the dataset. Shi: name in Shiwiar, Ki: name in Kichwa. (A) *Trachelyopterus galeatus*. (Shi: Puua). (B) *Hassar orestis*. (C) *Oxydoras niger* (Sh: Kuyukuyu, Ki: Turushuco). (D) *Pariolius armillatus* (Shi: Kumbautum). (E) *Pimelodella buckleyi* (Ki: Tugsig) (F) *Rhamdia quelen* (Shi: Kumbautum, Ki: Cumbarama).

**Identification.** Elongate body, robust, slightly depressed. Head: Broad, bare, unplated skin. Mouth: Subterminal, wide, with fleshy lips; three pairs of well-developed barbels: one long maxillary pair and two shorter mental pairs. Viliform teeth on premaxilla. Dorsal fin with a long, curved anterior hard ray followed by soft rays; developed adipose fin. Forked caudal fin. Overall coloration: blackish or dark brown dorsally, with a lighter brown belly. Large, irregular spots on the dorsolateral region. Fins without defined bands, with a dotted pattern.

*Hassar orestis:* **Ecuador:** MECN-DP 6372, 2, 182–191 mm SL.

Fig. 9B, (Table 2)

**Identification.** Body laterally compressed, elongated and fusiform. Mouth extensible, subterminal. Presence of curved odontodes distributed over the head and pectoral region, more developed in males. Dorsal fin with a serrated hard ray followed by soft rays. Body yellowish brown, with rounded dark spots in the opercular region and on the caudal peduncle. Presents a well-defined longitudinal black band that extends from the snout to the base of the caudal fin. Fins hyaline with orange tones and faint bands. Caudal fin forked.

*Oxydoras niger:* **Ecuador:** MECN-DP 6229, 1, 820 mm SL.

Fig. 9C, (Table 2)

**Identification.** A moderately large fish. It has simple maxillary and mental barbs, with eight branched rays on the lower lobe of the caudal fin. It has 20 to 26 plates on the lateral serrae. It is one of the largest species in the family. As its name indicates, its body is blackish, with yellowish tinges when it emerges from the water.

*Pariolius armillatus:* **Ecuador:** MECN-DP 6336, 4, 30–35 mm SL.

Fig. 9D, (Table 2)

**Identification.** Small fish. The body is elongated and laterally compressed, with a moderately long adipose fin. The head is dorsally flattened. The mouth is wide, in terminal position. Dark in color, with a white collar at the end of the head. The barbels reach halfway down the body.

*Pimelodella buckleyi:* **Ecuador:** MECN-DP 6268, 1, 77.2 mm SL; MECN-DP 6341, 1, 52 mm SL; MECN-DP 6417, 2, 208–240 mm SL; MECN-DP 6418, 1, 113 mm SL.

Fig. 9E, (Table 2)

**Identification.** Body elongated, slightly laterally compressed. Head depressed with large, lateral eyes. Mouth extensible, subterminal. It has three pairs of barbels: a long maxillary pair that reaches or exceeds the base of the pectoral fin and two shorter mental pairs. Dorsal fin with a serrated hard ray followed by soft rays. The body is yellowish-brown to greyish, with a well-defined, dark longitudinal band extending from the snout to the base of the caudal fin; it has no vertical bands. The fins are hyaline with light orange hues, without obvious bands. Caudal fin forked.

*Pimelodella gracilis*: **Ecuador:** MECN-DP 6284, 1, 105.2 mm SL.

(Table 2)

**Identification.** Elongated body, slightly compressed laterally towards the caudal region and somewhat depressed in the cephalic region, with a fusiform shape. The mouth is extensible, subterminal, and has three pairs of well-developed barbels. The body exhibits a general grayish or light brown coloration, with a well-defined longitudinal black band extending from the snout to the base of the caudal fin; it has no vertical bands. The fins are hyaline or slightly yellowish; occasionally, they have faint pigmentation on the margins. The caudal fin is slightly forked with rounded lobes. The dorsal fin has a serrated anterior hard ray, followed by soft rays.

*Pimelodella sp.:* **Ecuador:** MECN-DP 6342, 1, 50 mm SL.

(Table 2)

**Identification.** The body is elongated and slightly compressed laterally toward the caudal region. The head is depressed, with medium-sized lateral eyes. It has three pairs of well-developed barbels: one long maxillary pair and two shorter mental pairs. The body is yellowish-brown or light grayish, without bands. The fins are hyaline or yellowish in color, without visible bands. The caudal fin is forked.

*Rhamdia quelen*: **Ecuador:** MECN-DP 6349, 2, 90–100 mm SL; MECN-DP 6391, 1, 160 mm SL.

Fig. 9F, (Table 2)

**Identification.** A small to medium-sized fish, measuring around 14 cm in length. The body is moderately cylindrical. The eyes are dorsally positioned and moderately large. The head is moderately depressed, with a wide mouth. The caudal fin has upper and lower lobes of equal length. The coloration is brown with dark spots. The caudal fin is dark, with creamy bands on the outer edge. The bases of the dorsal, pectoral, and pelvic fins have a narrow creamy to yellowish band, while the remainder is dark. The adipose fin is very long and creamy-colored, with a thin dark band on the outer edge. The barbels of the upper jaw extend two-thirds of the fish’s length.

*Calophysus macropterus:* **Ecuador:** MECN-DP 6370, 1, 250 mm SL.

Fig. 10A, (Table 2)

**Figure 10:**
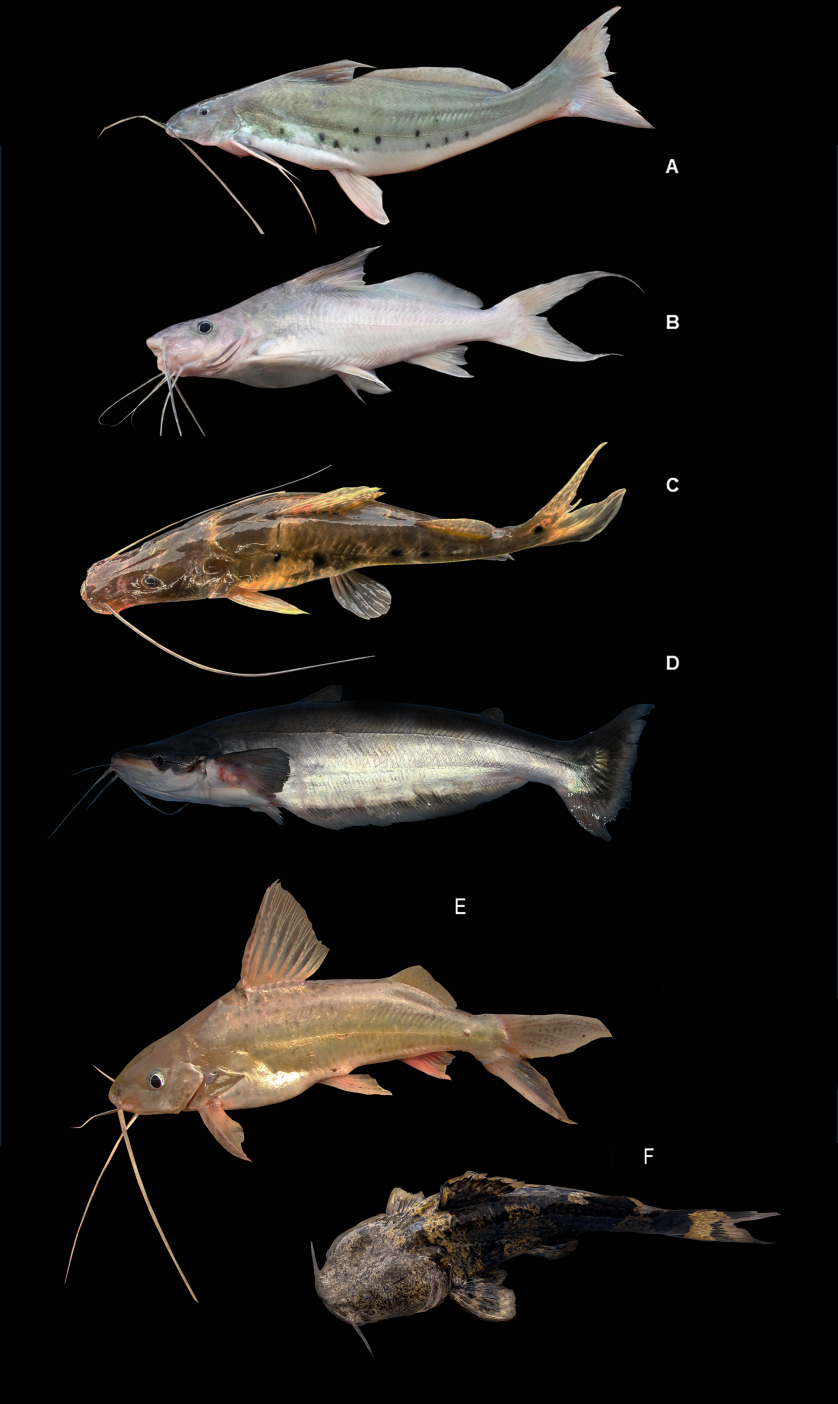
Representative Siluriformes of the Conambo River. Shiwiar and Kichwa names available are included as depicted in the dataset. Shi: name in Shiwiar, Ki: name in Kichwa. (A). *Calophysus macropt* (Shi: Tsake Muta, Ki: Yurak mota). (B) *Duopalatinus peruanus* (Shi: Karaimiur). (C) *Hemisorubim platyrhynchus* (Shi: Napi kungush). (D) *Hypophthalmus oremaculatus* (Shi: Unchiminiar Muuta, Ki: Maparachi). (E) *Pimelodus blochii* (Shi: Warrui, Ki: Boluquiqui). (F) *Microglanis zonatus* (Shi: Puua).

**Identification.** Medium-sized fish. Laterally compressed body, dorsally depressed head. Wide mouth, positioned terminally. Distinguished by its long adipose fin, pointed premaxillary teeth arranged in a single row, and the presence of black spots on the lateral flanks. Caudal fin emarginated.

*Cheirocerus eques*: **Ecuador:** MECN-DP 6368, 2, 150–160 mm SL.

(Table 2)

**Identification.** A moderately small to medium-sized fish. The mouth is ventrally positioned, with fleshy lips. The upper lip forms a deep pocket on each side. The eyes are laterodorsal and moderately large. The swim bladder has a curled edge, with a thin, elongated tube extending anterolaterally. The gill arches have fleshy papillae. It has between 17 and 21 gill arches. The adipose fin is moderately long. The barbels of the upper jaw are long, extending several centimeters beyond the outer tip of the caudal fin. The body is silvery to gray. It has a moderately wide black band that crosses the nape of the neck at the origin of the dorsal fin.

*Duopalatinus peruanus:* **Ecuador:** MECN-DP 6371, 1, 210 mm SL.

Fig. 10B, (Table 2)

**Identification.** It is a medium-sized fish, reaching around 20 cm in length. It is distinguished by its several rows of very small, narrow teeth. The upper jaw protrudes slightly above the lower jaw. The eyes are dorsally positioned and moderately large. The adipose fin is moderately long. The dorsal fin has six branched rays and a stiff spine. The barbels of the upper jaw are very long, almost a third longer than the length of the fish. The color is grayish to whitish, with yellowish fins.

*Hemisorubim platyrhynchos:* **Ecuador:** MECN-DP 6364, 1, 302 mm SL; MECN-DP 6365, 1, 300 mm SL.

Fig. 10C, (Table 2)

**Identification.** Medium-sized fish. It is distinguished by its very depressed and wide head, which is twice its height at the cleithrum. It has a wide mouth, with a prognathism of the lower jaw. The eyes are located laterodorsally. The nuchal plate and occipital process are connected. The dorsal fin has a rigid spine and six branched soft rays. The pectoral fins have one spine and seven soft rays. The color pattern is silvery to light gray in the laterodorsal region, with black ocelli. The fins are hyaline and also have ocelli. The ventral region is whitish.

*Hypophthalmus oremaculatus:* **Ecuador:** MECN-DP 6369, 1, 320 mm SL.

Fig. 10D, (Table 2)

**Identification.** Medium-sized species, reaching 39 cm in length. Laterally compressed body. Head moderately depressed towards the end of the snout. It is distinguished by its moderately forked caudal fin. It has 55 to 59 vertebrae. The mental barbels are long, sometimes extending beyond the origin of the pectoral fins and are ribbon-like. Moderately large, laterally positioned eyes. Very long anal fin, with 58 to 71 rays. Body silvery to light gray towards the ventral region. Hyaline fins with dark-colored outer edges.

*Pimelodus blochii:* **Ecuador:** MECN-DP 6420, 3, 140–181 mm SL.

Fig. 10E, (Table 2)

**Identification.** Small to medium-sized catfish. Distinguished by the highly ossified, pointed rays on the dorsal and pectoral fins. Body color is silvery to yellowish-gold, with hyaline fins. Mouth positioned terminally, pointing slightly downwards. Very long barbels. Caudal fin forked.

*Pseudoplatystoma punctifer:* **Ecuador:** MECN-DP 6230, 1, 820 mm SL.

(Table 2)

**Identification.** A large species, which can reach over 100 centimeters. The head is elongated and moderately flatenned, with moderatelly small eyes, positioned dorsally. The mouth is subterminal and wide. The coloration consists of black lines and dots alternating with white vertical lines. Fins covered in small black and round spots. Caudal fin forked.

*Sorubim elongatus:* **Ecuador:** MECN-DP 6352, 1, 235 mm SL.

(Table 2)

**Identification.** Medium-sized fish. Species of the genus Sorubim are distinguished by their long, highly depressed heads. The upper jaw is markedly prognathic. The eyes are laterally positioned. In this species, the upper and lower lobes of the caudal fin are the same size. The pectoral fins have eight soft rays, and the anal fin has between 21 and 22. The dorsal region is medium gray. The mental barbels extend more than halfway down the body. The ventral region is whitish. It has a wide, black lateral band extending from the snout to the tip of the lower lobe of the caudal fin.

*Microglanis zonatus:* **Ecuador:** MECN-DP 6275, 2, 33–49 mm SL.

Fig. 10F, (Table 2)

**Identification.** Small fish. Head broad and depressed. Body moderately laterally compressed. Mouth terminally positioned, with moderately long barbels reaching the tip of the humeral process. Mouth open 1.7 times the length of the head. Eyes small, dorsally positioned. Caudal fin emarginate to forked. Pelvic fins completely ventral. Caudal peduncle tall. The pectoral fin spines have very pronounced serrations on the outer edges. The body is sandy in color, with dark brown transverse bands that may merge ventrally. The fins have alternating dark and light bands.

*Aequidens tetramerus*: **Ecuador:** MECN-DP 6306, 1, 30.5 mm SL; MECN-DP 6340, 5, 30–45 mm SL; MECN-6343, 2, 19–22 mm SL.

Fig. 11A, (Table 2)

**Figure 11:**
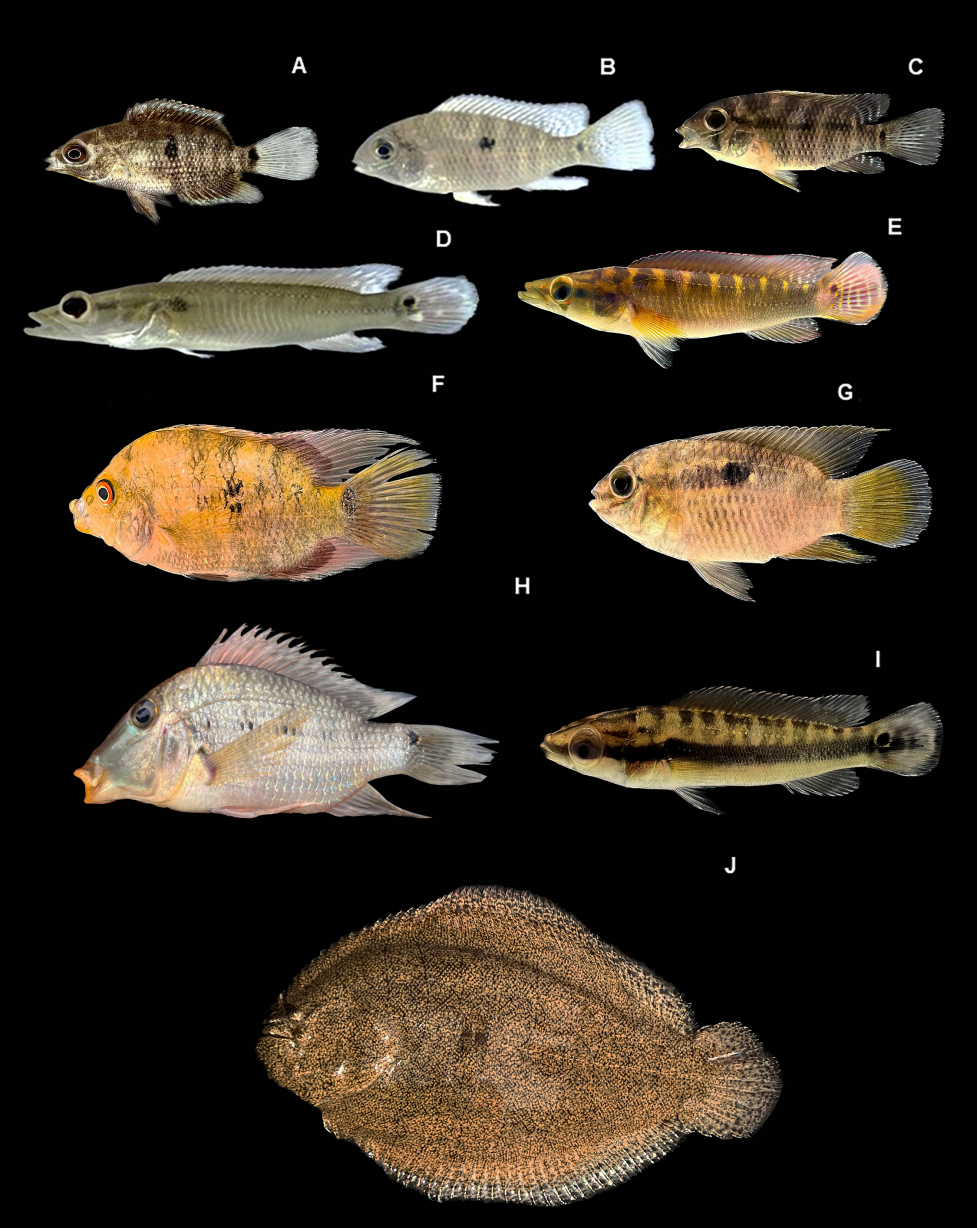
Representative fishes of the Conambo River. Shiwiar and Kichwa names available are included as depicted in the dataset. Shi: name in Shiwiar, Ki: name in Kichwa. (A) *Aequidens tetramerus* (Shi: Pakuy kandash, Ki: Uputasa). (B) *Bujurquina aff. huallagae* (C) *Bujurquina pardus* (Shi: Kandash). (D) *Crenicichla lucius* (Ki: Chuti). (E) *Crenicichla* aff. *sedentaria* (Shi: Chuwiu, Ki: Chuti). (F) *Heroina isonycterina* (Shi: Pakikunchum). (G) *Laetacara flavilabris* (Shi: Kandash). (H) *Satanoperca jurupari* (Shi: Pakimushu). (I) *Saxatilia proteus* (Shi: Chuwiu). (J) *Hypoclinemus mentalis* (Shi: Temash Tsarur).

**Identification.** An oval-bodied fish with a moderatelly long and tall caudal peduncle. It has no scales on the preopercle. The coloration pattern consists of three ocelli: a large, black ocellus at midbody, surrounded by a lighter golden-yellow color; another ocellus on the posterior region of the caudal peduncle, and another on the preopercle.

*Bujurquina aff. huallagae:* **Ecuador:** MECN-DP 6322, 1, 51 mm SL; MECN-DP 6350, 1, 50 mm SL.

Fig. 11B, (Table 2)

**Identification.** Body laterally compressed, oval and moderately elongated. Small head with slightly convex forehead. Interrupted lateral line, with clearly visible sensory pores. Extensible mouth, terminal. Long dorsal fin, with hard and soft rays. General coloration yellowish brown to greenish, with irregular spots in the opercular region. It has a well-defined dark longitudinal band that runs from the snout, through the eye and extends to the base of the caudal fin; hyaline pectoral fins, dorsal, anal and caudal fins with orange or reddish tones and transverse bands. Caudal fin rounded or slightly truncated.

*Bujurquina mariae*: **Ecuador:** MECN-DP 6280, 2, 30–54 mm SL.

(Table 2)

**Identification.** Small species, reaching about 10 cm in length. Laterally compressed, oval-shaped body. Oval head profile. The eyes represent 37% of the head length. The mouth is subterminal. The caudal peduncle covers between 15 and 16% of the standard length. Anal fin with 7–8 branched soft rays. Dorsal fin with 10–12 branched soft rays. The anterior lateral line has between 13 and 15 perforated scales, and the posterior between 9 and 12. The body is light-colored, between yellowish and greenish. It has 6 to 7 dark transverse bands, with an oblique longitudinal band that arises from the eye sockets and extends towards the posterior end of the dorsal fin. It also has a vertically elongated ocellus on the upper half of the caudal peduncle.

*Bujurquina pardus:* **Ecuador:** MECN-DP 6305, 1, 436 mm SL; MECN-DP 6314, 6, 42–78 mm SL.

Fig. 11C, (Table 2)

**Identification.** A small fish, not exceeding nine cm in length. The body is oval and moderately deep. The mouth is subterminal. The pectoral fins are short, representing 32% of the standard length. The cheeks show three rows of cycloid scales. The dorsal, anal, and pectoral fins are scaleless. The caudal fin is covered halfway by ctenoid scales, but not very densely. The caudal fin is truncated. It has 14 to 16 unicuspid conical teeth on the premaxilla. It can be distinguished from other species of the genus by the presence of a pattern of small squares at the central end of the scales. This is also distinguished because the band of the operculum is curved, and the third transverse band does not reach the lateral band.

*Bujurquina syspilus:* **Ecuador:** MECN-DP 6323, 1, 50 mm SL.

(Table 2)

**Identification.** Body laterally compressed, oval, moderately deep. Mouth extensible, terminal. Body light brown to yellowish in color, with a well-defined dark longitudinal band extending from the snout, through the eye, and reaching the base of the caudal fin; rounded spots are frequently observed on the opercular region and caudal peduncle. Dorsal fin long with hard and soft rays, slightly pointed posterior margin, rounded or slightly truncated caudal. Pectoral fins hyaline; dorsal, anal, and caudal fins with reddish, orange, or bluish pigmentation.

*Crenicichla lucius*: **Ecuador:** MECN-DP 6333, 1, 56 mm SL; MECN-DP 6334, 1, 70 mm SL; MECN-DP 6335, 1, 70 mm SL.

Fig. 11D, (Table 2)

**Identification.** Body elongated, fusiform and slightly laterally compressed. Head large, elongated, with a pointed snout. Mouth extensible, terminal. Dorsal fin long, with hard and soft rays, the posterior margin straight or slightly rounded. Body greenish-brown to yellowish, with a well-defined dark longitudinal band extending from the snout to the end of the caudal peduncle. Pectoral fins hyaline; dorsal, anal and caudal fins with reddish or orange hues, occasionally with diffuse transverse bands. Caudal fin rounded or slightly truncated.

*Crenicichla* aff. s*edentaria*: **Ecuador:** MECN-DP 6302, 3, 35.9–97.5 mm SL.

Fig. 11E, (Table 2)

**Identification.** Body elongated, fusiform and slightly laterally compressed. Head robust, straight profile, with a pointed snout and prominent lower jaw. Mouth extensible, terminal. Long dorsal fin with hard and soft rays, straight or slightly rounded posterior margin. Body greyish brown to greenish in colour, with a well-defined dark longitudinal band from the snout to the base of the caudal fin, with a rounded spot on the caudal peduncle. Pectoral fins hyaline; dorsal, anal and caudal fins with orange or reddish tones and, sometimes, with faint transverse bands. Caudal fin truncated or slightly rounded.

*Heroina isonycterina:* **Ecuador:** MECN-DP 6247, 7, 22–111 mm SL.

Fig. 11F, (Table 2)

**Identification.** Medium-sized species. The paired middle teeth of the lower jaw are short compared to those of the lower jaw. The body is deep. The species has a medial lateral ocellus and a smaller one on the upper region of the caudal peduncle, close to the caudal fin. It has several lighter, more diffuse vertical lines. The fins are hyaline.

*Heros efasciatus:* **Ecuador:** MECN-DP 6266, 1, 30 mm SL.

(Table 2)

**Identification.** Medium-sized fish. The body is tall and oval. The mouth is terminal and retractile. It has clear, diffuse vertical lines that become darker and more defined toward the ventral region, with the last one, before the beginning of the caudal peduncle, being darker in color. The species has iridescent spots on the lateral region. Males exhibit a deep orange coloration on the area of the body immediately posterior to the head.

*Laetacara flavilabris:* **Ecuador:** MECN-DP 6261, 1, 92.5 mm SL; MECN-DP 6262, 3, 373–535 mm SL; MECN-DP 6330, 1, 40 mm SL.

Fig. 11G, (Table 2)

**Identification.** A small fish with a rounded profile. It has a relatively long and deep, oval body. Mouth positioned terminally. It has two rows of scales on its cheeks and no ocellus on its caudal peduncle. It is distinguished by an oblique black band running from the end of the eye to the lateral median ocellus. It also has several clear vertical lines. Caudal fin round.

*Satanoperca jurupari:* **Ecuador:** MECN-DP 6367, 1, 135 mm SL.

Fig. 11H, (Table 2)

**Identification.** Medium-sized fish. It has an ocellus on the upper region of the caudal peduncle, a light brown lateral midline, and several less pronounced vertical bands, spots, and iridescent lines on the cheeks. The fins are hyaline. The dentition is uniseriate. The cheeks are bare. The mouth is in a lower terminal position.

*Saxatilia proteus*: **Ecuador:** MECN-DP 6270, 1, 60 mm SL; MECN-DP 6271, 1, 399 mm SL.

Fig. 11I, (Table 2)

**Identification.** Body elongated, fusiform, and slightly laterally compressed. Head broad, with a straight profile and a short, rounded snout. Overall coloration olive-brown to grayish, with a well-defined dark longitudinal band from the snout to the caudal peduncle. Opercular region with scattered dark spots. Pectoral fins hyaline; dorsal, anal, and caudal fins with orange or reddish hues, sometimes with faint transverse bands. Caudal fin truncated or slightly lobed.

*Hypoclinemus mentalis:* **Ecuador:** MECN-DP 6236, 1, 175 mm SL; MECN-DP 6374, 1, 260 mm SL.

Fig. 11J, (Table 2)

**Identification.** Small to medium-sized fish. Body is highly compressed laterally, but in adults, the left side becomes the ventral region. Relatively large eyes, located on the right side of the body. Oblique mouth. Villiform teeth arranged in patches. Sandy brown coloration with dark brown spots.

## Discussion

This dataset represents the first comprehensive compilation of fish species occurring in the Conambo River, a poorly documented tributary of the Tigre and Marañón rivers in the Ecuadorian Amazon. The records presented here substantially expand the ichthyological information available for this region and include several taxa that had not been previously reported from Ecuador, such as *Crenuchus spilurus* (Barriga, 2012), and specimens that could not be assigned to known species, suggesting the possible presence of undescribed taxa. By providing voucher photographs, collection codes, and precise locality data, this data resource establishes a reliable baseline for future taxonomic, biogeographic, and conservation research in the Marañón drainage.

Among the unidentified species, we collected a stingray *Potamotrygon* sp., which was only registered once (Fig. 4B). Furthermore, our datasets contribute to the knowledge of fish species in the Ecuadorian side of the Marañón drainage, which has been little explored. Most ichthyological inventories in the Ecuadorian Amazon focus on the northern area (Valdiviezo-Rivera, Carrillo-Moreno & Gea-Izquierdo, 2017; Tobes et al., 2022; Escobar-Camacho et al., 2025). Our results underscore the need for further ichthyological exploration in the Marañón drainage and highlight its importance for the conservation of freshwater fish diversity.

The fish species composition in the Conambo Basin reflects the history of tectonic activity, with the resulting uplift and fragmentation processes in the Marañón Drainage (Wesselingh Frank & Hoorn, 2011). These processes favored alternate periods of isolation and headwater stream capture from adjacent basins, which promoted both vicariance and geodispersal of fish species (Albert & Carvalho, 2011). For instance, the Pastaza Basin was intermittently connected to the Ucayali, promoting the mixing of fish faunas and increasing the species diversity in the Western Amazon (Lima & Ribeiro, 2011). These connections between the Pastaza and Ucayali basins resulted in shared species, such as *Steindachnerina guentheri* and *Cynodon gibbus*, while the presence of the genera *Creatrutus* and *Hemibrycon* derives from the Cis-Andean Foothills Pattern, which encompasses species adapted to fast-flowing, oxygen-rich streams along the Andean piedmont, and others such as *Curimatella meyeri, and Curimata aspera,* are related to the Amazon-Only Lowland Pattern, which are adapted to sediment-rich, nutrient-loaded whitewater rivers (Dagosta & De Pinna, 2019).

In the Marañón Drainage, most fish inventories have been conducted in Peru (Meza-Vargas et al., 2021; Ruiz-Tafur et al. , 2023). Within the Marañón Drainage, the ichthyofauna of the Ucayaly River has been studied in more detail (Chuctaya et al., 2022). In the Ucayali, fish species numbers surpass 150 (Rengifo, 2007; Aylas et al., 2023). Our results are comparable to records in waterbodies of the Pastaza River, where 108 species have been registered in the Zuñag and Anzu rivers (Galarza et al. , 2017). However, the fish assemblages in these rivers were dominated by species of high altitudes, adapted to fast flowing waters, belonging to the families Astroblepidae and Loricariidae, as well as by species of the genus *Bryconamericus*, of Characidae. In contrast, in the Conambo basin there were species typical of both low and moderately high altitudes, some of them typical of fast current streams (*e.g.*, genera *Creagrutus* or *Farlowella*) and others adapted to floodplains (*e.g.*, *Curimata aspera*, *Pseudoplatystoma punctifer*, or *Cichla monoculus*). The trophic structure of the fish assemblages in the Conambo Basin follows a trophic structure similar to other rivers in the Brazilian region of Western Amazon (Da Costa, Petry & Mazzoni, 2020): we observed a higher diversity of specialized invertivores of the genera *Hyphessobrycon* and *Moenkhausia* of *Characidae*, and *Microglanis* of Pseudopimelodidae, in forested streams and creeks, while generalist, omnivore species like the cichlid *Aequidens tetramerus*, were distributed in open areas. Unfortunately, the lack of information about the ichthyofauna of the Pindo and Tigre rivers hinders the comparisons of the ichthyofauna with these locations. Thus, our datasets can be used as a reference for the fish in these waterbodies and represent a baseline for the ichthyofauna of the Tigre Basin.

Characiformes and Siluriformes exhibited the highest numbers of species. At the family level, the assemblages Characidae showed the highest species numbers. These patterns are similar to other fish communities in the Amazon drainage (Van der Sleen & Albert, 2018). Among the Loricariidae of Siluriformes was the second richest family. This included species of the *Hypostomus cochliodon* group (Armbruster, 2003), *Ancistrus* (Provenzano & Barriga-salazar, 2018), as well as *Peckoltia* among the Hypostominae subfamily (Armbruster, Vander Sleen & Lujan, 2017), and other genera of the Loricariinae family, such as *Loricaria* and *Loricariichthys*, among others (Covain & Van der Sleen, 2018).

As with many remote areas of the Western Amazon, fieldwork in the Conambo Basin remains logistically challenging. Sampling was limited to two hydrological seasons, and additional surveys across different habitats and time periods will likely increase the number of documented species. Despite these limitations, the dataset represents a significant contribution to the ichthyological knowledge of the region and provides a foundation for further research, monitoring, and conservation planning.

The datasets also include information on species captured by Shiwiar and Sapara fishers, including several species of high subsistence value. The species reported as part of the fisheries include *Calophysus macropterus, Pseudoplatystoma punctifer*, and *Pimelodus blochii*, as well as Characiformes such as *Hoplias malabaricus, Rhaphiodon vulpinus*, *Mylossoma albiscopum, Hemiodus unimaculatus, Leporinus friderici, Schizodon fasciatus*, *Potamorhina latior, Prochilodus nigricans*, and *Brycon melanopterus*, and Cichlids like *Aequidens tetramerus, Crenicichla lucius, Heros efasciatus*, and *Satanoperca jurupari*. These fish are caught mainly with hooks and barbasco (*Lonchocarpus utilis*). These species are of great importance in both subsistence and commercial fisheries in the Amazon (Jácome-Negrete, 2013; Jácome-Negrete, Torres Jimenez & Guarderas Flores, 2022). These records highlight the cultural and food-security relevance of the ichthyofauna of the Conambo River and provide additional context regarding locally important fisheries resources. Although these data do not represent a systematic assessment of fisheries, they complement specimen-based records and may serve as reference information for future studies on fishing practices in Indigenous territories.

By making these records publicly available, we aim to facilitate their integration into broader scientific efforts, including taxonomic revisions, biodiversity assessments, environmental impact studies, and future conservation initiatives in the Marañón drainage. We hope the records provided here, which include coordinates, habitats, and hydrological seasons, as well as the vernacular names of the species, may serve as a baseline for further exploration of the fish fauna of Central Ecuadorian Amazon. We also hope that the information provided in this article can be used as a basis for the development of fishing agreements by the Shiwiar and Zápara communities of the Conambo River.

## Conclusions

This data report provides the first consolidated record of freshwater fish species from the Conambo River Basin, including voucher specimens, locality data, photographic documentation, and complementary ecological information such as hydrological season and habitat type. The dataset documents several species not previously reported for Ecuador and includes specimens that may represent undescribed taxa, offering an essential baseline for future taxonomic and biogeographic research in the Marañón drainage. In addition, the inclusion of species captured by Shiwiar and Sapara fishers provides a valuable reference for understanding locally important fish resources and their subsistence use in Indigenous territories. Although further sampling will likely expand the species list, the datasets made available here constitute a robust foundation for subsequent studies on freshwater biodiversity, habitat associations, and fisheries-related research in this understudied region of the Ecuadorian Amazon.

## Supplemental Information

10.7717/peerj.21003/supp-1 Supplemental Information 1 Taxonomic information, locality details, and basic metadata for each fish specimen record Information about the collection details of the fish specimens registered in the basin of the Conambo River.

10.7717/peerj.21003/supp-2 Supplemental Information 2 Metadata of Conambo_DBC dataset

10.7717/peerj.21003/supp-3 Supplemental Information 3 Fisheries practices by the Shiwiar and Zápara Communities of the Conambo River Information about the fishing practices of the Shiwiar and Zápara communities located in the Conambo River, obtained through interviews.

10.7717/peerj.21003/supp-4 Supplemental Information 4 Metadata of the dataset Conambo_DB_fisheries

10.7717/peerj.21003/supp-5 Supplemental Information 5 Supplementary Figure 1. Ancestral fishing technique using Barbasco (A) Roots of Lonchocarpus utilis. (B) A fisherman places the pack of roots in the stream. (C) Two fishers enclose thesegment of the stream with gill nets. (D) Fish are caught with hand nets.

10.7717/peerj.21003/supp-6 Supplemental Information 6 Code to generate the accumulation curves and other accompanying figures

10.7717/peerj.21003/supp-7 Supplemental Information 7 Data used to generate the accumulation curves and accompanying figures

## Additional Information and Declarations

### Field Study Permissions

The following information was supplied relating to field study approvals (i.e., approving body and any reference numbers): Field collections were approved by the Ministerio de Ambiente y Agua MAATE-ARSFC-2023-2996 and MAATE-ARSFC-2024-0411

### Data Availability

The following information was supplied regarding data availability: The raw data and code are available in the Supplemental Files.
