# Supplementary material for: Fishes of the Conambo River Basin, an unexplored area in the Ecuadorian Amazon: first annotated checklist
Source: PeerJ. 2026 Mar 24;14:e21003. doi: 10.7717/peerj.21003 (PMC13024240; doi:10.7717/peerj.21003)
Supplement: Supplemental Information 5 — (A) Roots of Lonchocarpus utilis. (B) A fisherman places the pack of roots in the stream. (C) Two fishers enclose thesegment of the stream with gill nets. (D) Fish are caught with hand nets. [file peerj-14-21003-s005.pptx]

## Slide 1
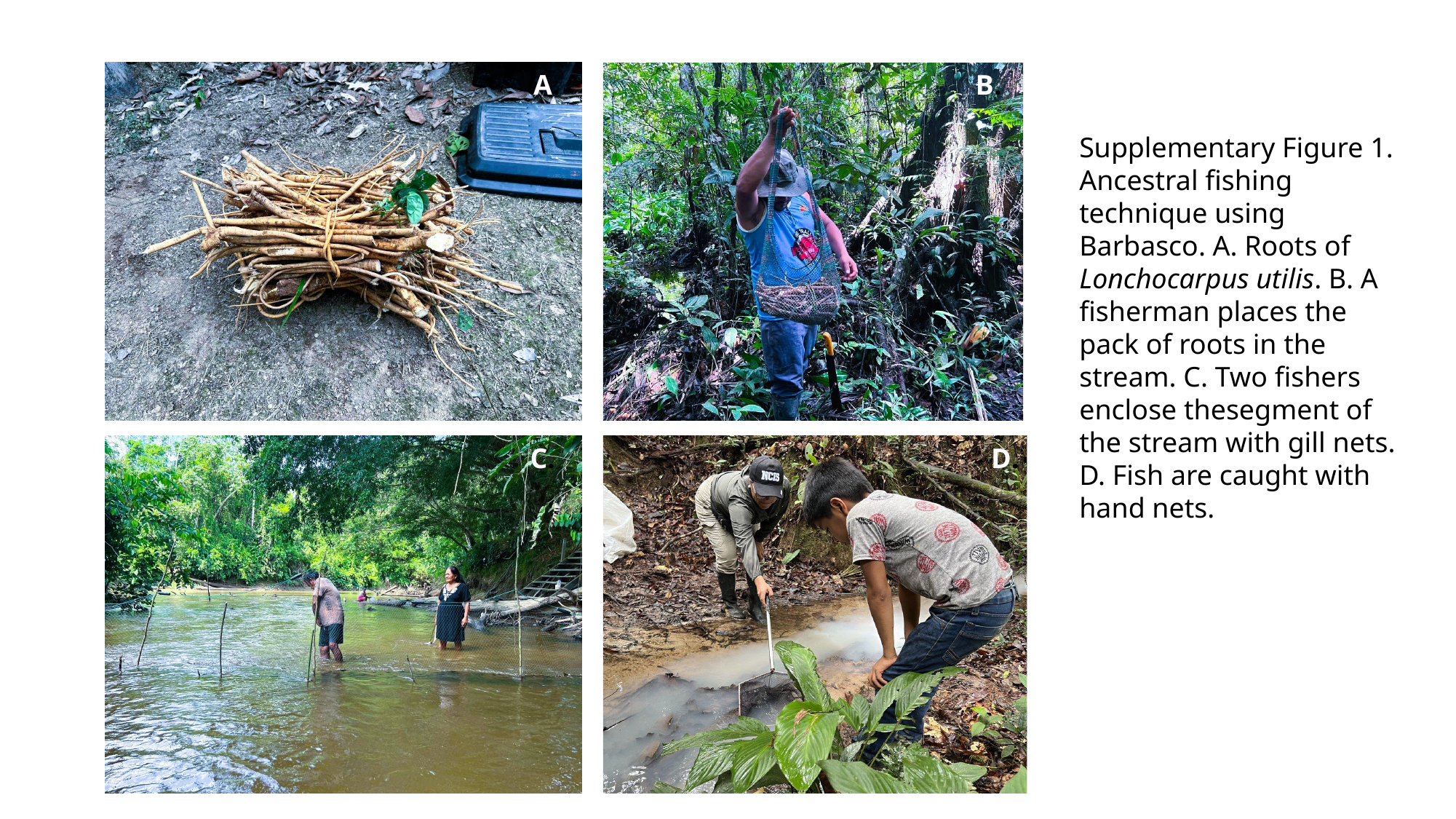

A.
B.
Supplementary Figure 1. Ancestral fishing technique using Barbasco. A. Roots of Lonchocarpus utilis. B. A fisherman places the pack of roots in the stream. C. Two fishers enclose thesegment of the stream with gill nets. D. Fish are caught with hand nets.
C.
D.
